# Propionic acid promotes neurite recovery in damaged multiple sclerosis neurons

**DOI:** 10.1093/braincomms/fcae182

**Published:** 2024-06-03

**Authors:** Barbara Gisevius, Alexander Duscha, Gereon Poschmann, Kai Stühler, Jeremias Motte, Anna Lena Fisse, Sanja Augustyniak, Adriana Rehm, Pia Renk, Celina Böse, Diana Hubert, Kathrin Peters, Michelle Jagst, André Gömer, Daniel Todt, Verian Bader, Marianne Tokic, Sarah Hirschberg, Christos Krogias, Nadine Trampe, Charlotta Coutourier, Carmen Winnesberg, Eike Steinmann, Konstanze Winklhofer, Ralf Gold, Aiden Haghikia

**Affiliations:** Department of Neurology, St. Josef Hospital, Ruhr-University Bochum, 44791 Bochum, Germany; Department of Neurology, St. Josef Hospital, Ruhr-University Bochum, 44791 Bochum, Germany; Department of Neurology, Otto-von-Guericke University, 39120 Magdeburg, Germany; Institute of Molecular Medicine, Proteome Research, Medical Faculty and University Hospital, Heinrich Heine University Düsseldorf, 40225 Düsseldorf, Germany; Molecular Proteomics Laboratory, BMFZ, Heinrich Heine University Düsseldorf, 40335 Düsseldorf, Germany; Institute of Molecular Medicine, Proteome Research, Medical Faculty and University Hospital, Heinrich Heine University Düsseldorf, 40225 Düsseldorf, Germany; Molecular Proteomics Laboratory, BMFZ, Heinrich Heine University Düsseldorf, 40335 Düsseldorf, Germany; Department of Neurology, St. Josef Hospital, Ruhr-University Bochum, 44791 Bochum, Germany; Department of Neurology, St. Josef Hospital, Ruhr-University Bochum, 44791 Bochum, Germany; Department of Neurology, St. Josef Hospital, Ruhr-University Bochum, 44791 Bochum, Germany; Department of Neurology, St. Josef Hospital, Ruhr-University Bochum, 44791 Bochum, Germany; Department of Neurology, St. Josef Hospital, Ruhr-University Bochum, 44791 Bochum, Germany; Department of Neurology, St. Josef Hospital, Ruhr-University Bochum, 44791 Bochum, Germany; Department of Neurology, St. Josef Hospital, Ruhr-University Bochum, 44791 Bochum, Germany; Department of Neurology, St. Josef Hospital, Ruhr-University Bochum, 44791 Bochum, Germany; Department for Molecular and Medical Virology, Ruhr-University Bochum, 44801 Bochum, Germany; Institute of Virology, University of Veterinary Medicine Hannover, 30559 Hannover, Germany; Department for Molecular and Medical Virology, Ruhr-University Bochum, 44801 Bochum, Germany; Department for Molecular and Medical Virology, Ruhr-University Bochum, 44801 Bochum, Germany; European Virus Bioinformatics Center (EVBC), 07743 Jena, Germany; Department of Molecular Cell Biology, Institute of Biochemistry and Pathobiochemistry, Ruhr-University Bochum, 44801 Bochum, Germany; Department of Medical Informatics, Biometry and Epidemiology, Ruhr University Bochum, 44780 Bochum, Germany; Department of Neurology, St. Josef Hospital, Ruhr-University Bochum, 44791 Bochum, Germany; Department of Neurology, St. Josef Hospital, Ruhr-University Bochum, 44791 Bochum, Germany; Department of Neurology, St. Josef Hospital, Ruhr-University Bochum, 44791 Bochum, Germany; Department of Neurology, St. Josef Hospital, Ruhr-University Bochum, 44791 Bochum, Germany; Department of Neurology, St. Josef Hospital, Ruhr-University Bochum, 44791 Bochum, Germany; Department for Molecular and Medical Virology, Ruhr-University Bochum, 44801 Bochum, Germany; Department of Molecular Cell Biology, Institute of Biochemistry and Pathobiochemistry, Ruhr-University Bochum, 44801 Bochum, Germany; Cluster of Excellence RESOLV, 44801 Bochum, Germany; Department of Neurology, St. Josef Hospital, Ruhr-University Bochum, 44791 Bochum, Germany; Department of Neurology, St. Josef Hospital, Ruhr-University Bochum, 44791 Bochum, Germany; Department of Neurology, Otto-von-Guericke University, 39120 Magdeburg, Germany

**Keywords:** neurite recovery, neurodegeneration, gut microbiome metabolites, microbiome–gut–brain axis

## Abstract

Neurodegeneration in the autoimmune disease multiple sclerosis still poses a major therapeutic challenge. Effective drugs that target the inflammation can only partially reduce accumulation of neurological deficits and conversion to progressive disease forms. Diet and the associated gut microbiome are currently being discussed as crucial environmental risk factors that determine disease onset and subsequent progression. In people with multiple sclerosis, supplementation of the short-chain fatty acid propionic acid, as a microbial metabolite derived from the fermentation of a high-fiber diet, has previously been shown to regulate inflammation accompanied by neuroprotective properties. We set out to determine whether the neuroprotective impact of propionic acid is a direct mode of action of short-chain fatty acids on CNS neurons. We analysed neurite recovery in the presence of the short-chain fatty acid propionic acid and butyric acid in a reverse-translational *disease-in-a-dish* model of human-induced primary neurons differentiated from people with multiple sclerosis-derived induced pluripotent stem cells. We found that recovery of damaged neurites is induced by propionic acid and butyric acid. We could also show that administration of butyric acid is able to enhance propionic acid-associated neurite recovery. Whole-cell proteome analysis of induced primary neurons following recovery in the presence of propionic acid revealed abundant changes of protein groups that are associated with the chromatin assembly, translational, and metabolic processes. We further present evidence that these alterations in the chromatin assembly were associated with inhibition of histone deacetylase class I/II following both propionic acid and butyric acid treatment, mediated by free fatty acid receptor signalling. While neurite recovery in the presence of propionic acid is promoted by activation of the anti-oxidative response, administration of butyric acid increases neuronal ATP synthesis in people with multiple sclerosis-specific induced primary neurons.

## Teaser

In human multiple sclerosis-specific neurons, differentiated via induced pluripotent stem cells, Gisevius *et al*. display neuroregeneration mediated by the short-chain fatty acids propionic and butyric acid. Intracellularly, free fatty acid receptor signalling leads to inhibition of histone deacetylase activity, thereby altering the oxidative stress response and cellular protein biosynthesis.

## Graphical Abstract

**Graphical Abstract:**
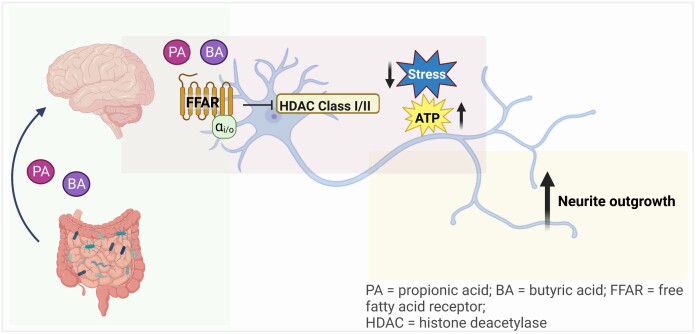

## Introduction

Progressive accumulation of neurodegeneration still constitutes the hallmark of the autoimmune-mediated disease multiple sclerosis, affecting 2.5 million people worldwide, mostly manifesting in young women at the ages of 18–30.^1^ Focal inflammation within the CNS results from infiltrating autoreactive lymphocytes following breakdown of the blood–brain barrier (BBB), causing demyelination of axons. Among others, reactive oxygen species constitutes a main driver of neuroinflammation, leading to irreversible neuronal loss and progressive neurodegeneration.^2,3^

Currently available disease-modifying treatments (DMTs) are aimed to target the immunomodulatory properties of multiple sclerosis and are therefore mainly effective in the relapsing–remitting (RRMS) form of the disease, approximately affecting 80% of people with multiple sclerosis (pwMS).^4^ However, treatment options for progressive forms remain sparse, with the majority of RRMS cases converting into a secondary progressive (SPMS) form after 15–20 years and 10–15% of pwMS starting with a primary progressive (PPMS) form already at disease onset.^4-6^ Since therapeutic options are not able to prevent both ongoing neurodegeneration and the conversion into the progressive form, it remains an open question if multiple sclerosis is a rather primary neurodegenerative disorder^7,8^ or if the progressive form results from an exhaustion of regenerative capacities of the CNS.^9^

Diet and the gut microbiome have evolved as potential risk factors in the aetiology of multiple sclerosis,^10^ participating into disease manifestation as well as exacerbation of clinical symptoms.^11,12^ As main bacterial metabolites, derived from the fermentation of a high-fiber diet by the commensal gut microbiota,^13^ short-chain fatty acids (SCFAs) are involved in various physiological processes, among them orchestrating the polarization and differentiation of immune cell subsets.^14^ Thus, by constantly challenging the immune compartment, bacterial metabolites ensure the crosstalk between the residing commensals and the subepithelial, gut-associated lymphatic tissue (GALT).^15^ Hence, malfunction of this equilibrium, i.e. by a dysbiotic state of the microbiome, is hypothesized to give rise to the development and exacerbation of multiple sclerosis.

Previously, we and others demonstrated that pwMS display deficiencies in faecal and systemic SCFA levels,^16-18^ associated with a dysbiotic microbiome,^19,20^ particularly at the time of disease manifestation. In a translational approach, we could show that administration of the SCFA propionic acid (PA) in pwMS leads to an alleviation of disease activity and progression by adjusting anti-inflammatory Treg cell levels and function. Clinically, longitudinal PA supplementation in pwMS led to volume increase of certain brain areas, predominantly of the basal ganglia. This was accompanied by an increased PA availability in the CSF.^16^ We had previously observed reduced axonal loss in the spinal cord tissue of the experimental model of multiple sclerosis under PA^21^; thus, the question arose whether PA may exert neuroprotection indirectly, by reducing neuroinflammation, or directly by modifying the neuroregenerative capacities of the CNS.^22^

To investigate the putative direct neuroprotective role of SCFA in the context of multiple sclerosis, in this project, we used a reverse-translational model of induced primary neurons (iPNs), generated from induced pluripotent stem cells (iPSCs) of pwMS.^23^ Since glial- and CNS-residing immune cells are absent in this model, this methodology enables the possibility to examine exclusively multiple sclerosis-associated neurodegenerative aspects, which are otherwise difficult to assess, compared with the immunomodulatory features of the disease.^24^

We found that the SCFAs PA and BA improved neuroregeneration in pwMS-specific iPNs, mediated by free fatty acid receptor (FFAR) signalling and inhibition of histone deacetylase (HDAC) class I/II activity.

## Materials and methods

### Patients and ethical statement

This study was approved by the ethics committee of the Department of Medicine at the Ruhr University Bochum (Reg Nr. 4493–12). Participants included into this study were affiliated with St. Josef-Hospital, University Hospital of Ruhr University in Bochum. Following written consent, 200 ml urine, two EDTA, and two serum samples were collected from study participants. Of the pwMS (*N* = 3) whose biological material was used in this study, at inclusion two were male (age 33) and one female (age 36), diagnosed with relapsing–remitting multiple sclerosis with an expanded disability status scale (EDSS) of 2.

### Evoked visual potentials

Visually evoked potential (VEP) data were collected from the occipital midline referred to a mid-frontal electrode according to the International Society for Clinical Electrophysiology of Vision standards (Keypoint.net, Neurolite Software, Natus, Switzerland). Pattern reversal VEP was produced by high-contrast, black and white checks. Each check has the size of 171.6 arc minute. The examination was performed in a dark room in a 1 m distance. P100 latency and the P100-P125 amplitude were collected. Patients were observed for approximately 4 years of follow-up.

### Generation of induced primary neurons

iPNs were generated from pwMS’ renal proximal tubule epithelial cells (RPTECs) as previously described.^23^ In brief, RPTECs were isolated from urine samples of pwMS and cultivated in Renal Epithelial Cell Growth Medium (REGM)^TM^ (#CC-3190, Lonza). Following cultivation, cells were reprogrammed to iPSCs by electroporation of the episomal plasmids (Addgene) pCXLE-hOCT3/4-shp53 (#27077), pCXLE-hSK (#27078), and pCXLE-hUL (#27080)^25^ via the Neon® Transfection System (Life Technologies) per manufacturer’s instruction. Afterwards, cells were cultivated in a TeSR™-E7™ medium (#05914, STEMCELL Technologies) until the formation of iPSC colonies. IPSC colonies were cultivated in a mTeSR™1 medium (#85850, STEMCELL Technologies). For embryoid body (EB) formation, respective iPSC colonies were scratched from plates manually, transferred onto non-coated dishes, and cultivated in a mTeSR™1 medium. To inhibit the formation of mesoderm and entoderm, the medium was supplemented with 10 µM SB431542 (#FBM-10-2443, BIOZOL) and 5 µM dorsomorphin (#866405-64-3, Sigma) the second day. Every other day, medium change was performed, and supplements were added freshly. At Day 6, EBs were transferred onto 0.002% poly-L-ornithine (PORN, #27378-49-0, Sigma)/10 µg/mL laminin (#114956-81-9, Sigma)-coated dishes and cultivated in a neural stem cell medium (NSCM) medium,^26^ composed of Dulbecco’s modified Eagle’s medium (DMEM)/F12 GlutaMAX™ (#31331028, Thermo Fisher Scientific), 20 µg/mL insulin (#91077C, Sigma), 1.6 g/l L-glucose (AppliChem), 1 µL/mL B-27™ supplement (#17504-044, Life Technologies), 1 µL/mL *N*-2 supplement (#17502048, Life Technologies), 10 ng/mL basic fibroblast growth factor (bFGF) (#1102021, PAN-Biotech), and 10 ng/mL epidermal growth factor (EGF) (#1101001, PAN-Biotech). When neural rosettes were formed, structures were cut manually and trypsinized for the isolation of neural precursor cells (NPCs). NPCs were cultivated in the NSCM medium. For differentiation of NPCs to iPNs, cells were trypsinized, seeded onto PORN-/laminin-coated dishes, and cultivated in the neuronal medium composed of DMEM/F12 GlutaMAX™, 2× *N*-2 supplement, 2× B-27™ supplement, 50 µg/mL apo-transferrin (#T8158, Sigma), and 200 µg/mL L-ascorbic acid (#3525.1, Carl Roth). For induction of differentiation, the medium was supplemented with 500 ng/mL sonic hedgehog (#100-45, PeproTech) and 4 µM retinoic acid (#R2625, Sigma) for the first 6 days. To ensure iPN maturation, on Day 7, supplements were changed to 10 ng/mL brain-derived neurotrophic factor (BDNF, #450-02, PeproTech) and 20 ng/mL glial cell-derived neurotrophic factor (GDNF, #450-10, PeproTech). IPNs were cultured at 37°C and 5% CO_2_ if not stated otherwise.

### RNA sequencing

RNA was isolated using the NucleoSpin RNA kit (#740955, MACHEREY-NAGEL) according to the manufacturer’s instructions. Sequencing libraries were prepared using the NEBNext Ultra II Directional RNA Library Prep Kit, and sequencing was conducted on the Illumina NovaSeq 6000 platform in 50-mer in paired end mode. Quality control, mapping against the human genome (Hg38), and statistical analysis for gene expression were conducted in CLC Genomics Workbench 22.0. Gene expression was calculated for individual transcripts as reads per kilobase per million bases mapped (RPKM). Data visualization was done in R with in-house scripts using the Tidyverse library and ComplexHeatmap.

### Neurite outgrowth assay

IPNs were seeded at 20 000–25 000 cells per well onto a 24-well plate previously provided with PORN-/laminin-coated coverslips. A neuronal medium was used as a cultivation medium. For neurite regrowth assay, the following day, iPNs were treated with 10 µM of the microtubule-destabilizing adjuvant nocodazole (#487928, Merck Millipore) for 6 h. Afterwards, the neuronal medium supplemented with nocodazole was withdrawn and replaced with the neuronal medium without nocodazole to induce neurite recovery. To investigate the impact of SCFA on neurite recovery, the neuronal medium was either supplemented with respective concentrations of PA (#P1880, Sigma), BA (#B5887, Sigma), or in combined treatment with 100 µM PA together with respective BA concentrations. Recovery assays were conducted for 24 h at 37°C and 5% CO_2_ in aqua dest. served as the control treatment. The measurement of the sum length of the neurite length was performed following β-III tubulin staining as described below. To calculate the neurite length, the sum length of all neurites per neuron was measured. The outcome ‘neurite length’ is the sum length of neurites per neuron. The neurite length was calculated by ImageJ/Fiji together with the Neuron J plugin.^27^

### Neurite outgrowth inhibitory assays

For inhibition of the FFAR Gα_i/o_ subunit, pertussis toxin (PTX, # P2980, Sigma) was used as specific antagonist. Neurite recovery assay was performed as previously described. Following neuronal damage, the medium was replaced and supplemented with 250 ng PTX in respective conditions; other conditions received PBS. Preincubation with PTX was performed for 30 min; afterwards the medium was exchanged and supplemented with respective SCFA concentrations, and neurite recovery assay was conducted for 24 h, as previously described.

The same procedure was conducted for inhibition of monocarboxylate transporter 1 (MCT1), by administration of the antagonist AZD3965 (#HY-12750, MedChemExpress). Since the inhibitor was diluted dimethyl sulfoxide (DMSO), 0.01% DMSO control was added to the experimental setup. Neurite recovery and measurement was performed as previously described.

### Immunofluorescent staining

Cells were washed once with 1× Dulbecco’s phosphate-buffered saline (DPBS) and fixed with 100% ice-cold methanol for 5 min. Permeabilization was performed by incubation with 0.3% Triton X-100 in 1× PBS for 3 min following another three washing steps with 1× PBS. Afterwards, cells were washed again three times with 1× PBS. Blocking of unspecific binding sites was performed by incubation with 5% bovine serum albumin (BSA) (#8076.4, Carl Roth) in 1× PBS for 1 h at room temperature (RT). Primary antibody incubation was performed overnight at 4°C in the dark [mouse anti-β-III tubulin, dilution 1:200 000, BioLegend (#657402; rabbit anti-FFAR 2, dilution 1:200, Antikörper-online.de, #ABIN6257801; rabbit anti-FFAR 3, dilution 1:100, Thermo Fisher Scientific, #PA5-75521: mouse anti-MCT1, dilution 1:500, Genetex, #GTX631643)] diluted in 0.8% BSA in PBS. The following day, cells were washed three times with 1× PBS and subsequently incubated with the secondary antibody [(goat anti-mouse IgG (H + L), Alexa Fluor 488, dilution 1:1000, Thermo Fisher Scientific, #A-11001; goat anti-rabbit IgG (H + L), Alexa Flour 568, dilution 1:1000, Thermo Fisher Scientific, #A-11036; goat anti-mouse F(ab’) 2 Fragment, Alexa Flour 555, dilution 1:1000, Life Technologies, #A-21425) diluted in 0.8% BSA in PBS for 2 h at RT in the dark. Following incubation, three washing steps with 1× PBS were conducted, and coverslips were mounted in DAPI Fluoromount-G (#SBA-0100-20, BIOZOL). Fluorescence signals for measurements of the neurite length were captured with Olympus Microscope BX51 (Olympus), FFAR, and MCT1 staining with an Axio Observer microscope with an Axiocam 512 mono camera, a witch 20,000× resolution, respectively. Analysis of neurite sum length was conducted only for iPNs, which showed no connection to surrounding neurons. Per condition, five fields per coverslip were acquired and analysed.

### Proteomic analysis

For protein isolation, cells were washed with ice-cold PBS and placed onto ice for a minimum of 20 min. For harvesting, cells were rinsed with ice-cold PBS and collected in a 15 ml falcon tube. Falcons were then centrifuged at 450 g for 3 min at 4°C. The supernatant was discarded, and the cell pellet was subsequently frozen at −80°C until further analysis.

Sample preparation for proteome analyses was performed as previously described.^28^ Cell pellets were lysed in a lysis buffer (30 mM Tris-HCL; 2 M thiourea, 7 M urea, 4% CHAPS (w/v) pH 8-0 in aqua dest.) and 5 µg of lysates shortly separated in a polyacrylamide gel (about 5 mm running distance). Afterwards, the gel was silver-stained and protein-containing bands were destained. Proteins were reduced with dithiothreitol and subsequently alkylated with iodoacetamide and digested by trypsin overnight. Peptides were finally resuspended in 0.1% trifluoroacetic acid. By using an UltiMate 3000 rapid separation liquid chromatography system (RSCL, Thermo Fisher Scientific), 500 ng of peptides mixture was separated by a 2 h gradient. Separated peptides were injected afterwards in an Orbitrap Elite high-resolution mass spectrometer (Thermo Fisher Scientific) operated in a positive mode via a nano-source electrospray interface (source voltage 1.5 kV). Survey scans are carried out in the Orbitrap analyser at a resolution of 60 000 (at 400 m/z), with a target value for automatic gain control of 1 000 000 and a maximum fill time of 200 ms. Isolation of the 20 most intense peptide ions with a minimal signal intensity of 500, charge state > 1, was performed. Afterwards, peptide ions were transferred to the linear ion trap (LTQ) part of the instrument and fragmented using collision-induced dissociation (CID). Peptide fragment analyses were conducted with a maximum fill time of 100 ms and an automatic gain control value of 10 000 at a resolution of 5400 (at 400 m/z). Fragmented ions were excluded from fragmentation for 45 s. Identification and quantification of proteins and peptides were performed in the MaxQuant environment (version 1.6.1.0, MPI for Biochemistry, Planegg, Germany) with standard parameters if not otherwise stated. Searches were carried out using 71 567 entries from the UP000005640 proteome dataset downloaded from UniProtKB on 28 August 2017. Carbamidomethyl was considered as fixed, and methionine oxidation and *N*-terminal acetylation were considered as variable modifications and the match between tuns option was enabled.

Proteins and peptides were accepted with a false discovery rate of 1%, and only proteins showing at least two different identified peptides were considered for further analysis. Data analysis was performed with Perseus (version 1.6.1.1., MPI for Biochemistry, Planegg, Germany). Protein entries were kept showing at least four valid values in one group (CTRL-Recovery/PA-Recovery). For statistical and categorical enrichment analysis, normalized label-free quantification intensities were log2-transformed and missing values imputed with values from a downshifted (1–8 standard deviations) normal distribution (width: 0.3 standard deviations). One-dimensional annotation enrichment analysis was conducted on protein-associated annotations [Gene Ontology molecular function (GOMF), Gene Ontology biological processes (GOBP) slim, Gene Ontology cellular components (GOCC) slim, = Kyoto Encyclopedia of Genes and Genomes (KEGG) name]. The given *q*-values were calculated from *P*-values by the method of Benjamini and Hochberg.^29^

### Quantitative real-time polymerase chain reaction

For quantitative reverse transcription polymerase chain reaction (qRT-PCR) analyses, pwMS-specific iPNs were isolated after 6 h recovery from the previous nocodazole damage. The total RNA was prepared from harvested cells via the RNeasy Mini Kit (250) (Qiagen) per manufacturer’s instructions, and 1 µg of the total RNA was transcribed into cDNA via the qScript™ cDNA Supermix (Promega) according to the manufacturer’s protocol.

For analysis, 100 ng cDNA was used in duplicates with specific primers (Table 1). Cycling conditions were 3 min 95°C; 15 s 95°C; 1 min 60°C; 15 s 95°C; 1 min 60°C; 30 s 95°C, and 1 min 60°C, while each reaction included 40 cycles. The qRT-PCR was performed with the QuantStudio™ 3 real-time PCR system by using the GoTaq® qPCR master mix coupled with the fluorescence dye SYBR Green.

**Table 1:** Primer for qRT-PCR experiments

Target gene	Forward	Reverse
Nrf2	GCGACGGAAAGAGTATGAGCT	GGCTGGCTGAATTGGGAGAA
NQO1	AGTCCATCCCAACTGACAACC	AAAGCAAGTCAGGGAAGCCT
GCL	AACCTCTGAACACCCAAGCC	GGCTGAGGAAAGATGTGGGG
GSR	GGACTTGGGTGTGATGAAATGC	GAGGTAGGGTGAATGGCGAC
TBP	GAGCTGTGATGTGAAGTTTCC	TCTGGGTTTGATCATTCTGTAG
GAPDH	GGCTCCCACCTTTCTCATCC	ACATCACCCCTCTACCTCCC

GAPDH, glyceraldehyde-3-phosphate dehydrogenase; GCL, glutamate–cysteine ligase; GSR, glutathione reductase; Nqo1, NAD(P)H quinone dehydrogenase 1; Nrf2, nuclear factor erythroid 2-related factor 2; TBP, TATA-box binding protein.

To normalize target gene expression levels, TATA-box binding protein (TBP) and GAPDH were used as housekeeping genes.

### Luminescence assays

For luminescent analysis, 35 000 cells per well were seeded onto a white 96-well plate (#353047, Corning). For ATP production analysis, iPNs were treated with different SCFA concentrations for 2 h, 4, 6, and 8 h. Aqua dest. was used as the control condition. Following treatment, the neuronal medium was removed and replaced by the CellTiter-Glo® 2.0 cell viability assay (#A2791, Promega), and subsequently the protocol was conducted per manufacturer’s instruction.

For HDAC activity assay, iPNs were treated with respective concentrations of PA and BA for 30 min. Analysis of HDAC class I/II inhibition was examined via the HDAC-Glo™ I/II Assay (#G6420, Promega) per manufacturer’s instructions.

Respective relative light units (RLUs) were measured by the Tecan Reader Infinite pro 200 (Thermo Fisher Scientific), using an integration time of 300 ms.

### Quantification and statistical statement

Statistical tests were performed by GraphPad Prism 6 and 10. Gaussian distribution was determined by D'Agostino–Pearson omnibus normality test with a confidence level of 95%. Applied statistical tests are provided within respective figure legends, the number of donors for the differentiation of iPNs is referred to as *N*, and independent experiments performed are referred to as *n*. If not stated otherwise, *n* represents the passages of NPCs differentiated to iPNs, which are used for the conduction of experiments. Statistical significance was indicated by one asterisk (*P* > 0.05), two asterisks (*P* > 0.01), and three asterisks (*P* > 0.001). Data are provided as mean ± SEM.

For analyses of the neurite length, statistical analyses were performed using R version 4.1.2 [R Core Team (2021). R Foundation for Statistical Computing, Vienna, Austria]. Fold change in the neurite length is reported as mean ± standard error of measurement (SEM). *n* denotes the number of neurons analysed. The outcome ‘neurite length’ is the sum length of neurites per neuron. All tests are preformed to α = 0.05. Differences in neurite length recovery between CTRL and experimental conditions are analysed via mixed linear regression models with added random effect for the cell donor. The estimated difference in neurite length recovery (estimate) with 95% confidence intervals (CIs) and *P*-values, derived by the Satterthwaite approximation,^30^ are reported.

## Results

### Propionic acid promotes neurite recovery of induced primary neurons via free fatty acid receptor signalling

**Figure 1:**
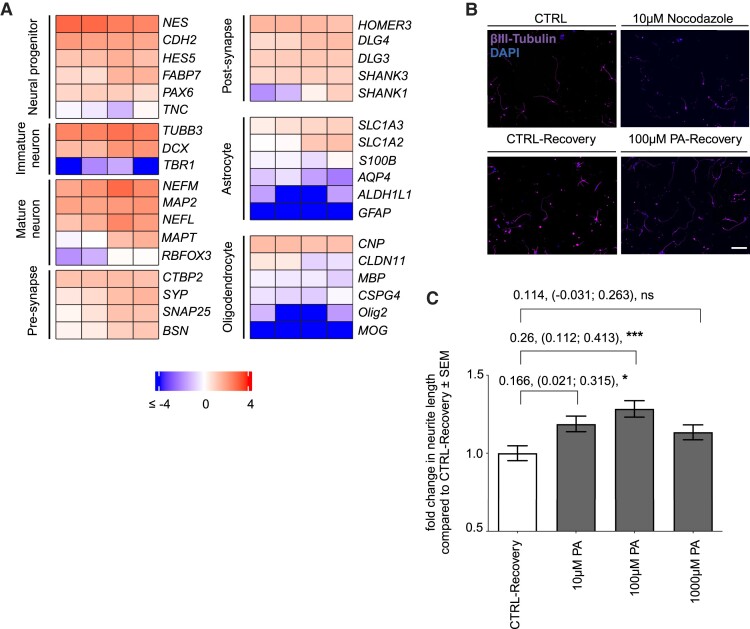
**PA promotes neuroregeneration.** (**A**) Characterization of pwMS-specific iPNs by RNA sequencing analyses at 4, 7, 12, 14, and 19 days of differentiation. Log10 of reads per kilobase of transcript per million mapped reads (RPKM) of the expression of neuron-specific markers is shown. Left column: Neural progenitors: Nestin (*NES*), cadherin 2 (*CDH2*), Hes family BHLH transcription factor 5 (*HES5*), fatty acid binding protein 7 (*FABP7*), paired box 6 (*PAX6*), tenascin C (*TNC*). Immature neurons: β-III tubulin (*TUBB3*), doublecortin (*DCX*), T-box brain transcription factor 1 (*TBR1*). Mature neurons: Neurofilament medium chain (*NEFM*), the dendritic marker microtubule-associated protein 2 (*MAP2*), neurofilament light chain (*NEFL*), microtubule-associated protein Tau (*MAPT*), RNA binding Fox-1 homologue 3 (*RBFOX3*). Pre-synapse: C-terminal binding protein 2 (*CTBP2*), synapse-specific marker synaptophysin (*SYP*), synaptosome-associated protein 25 (*SNAP25*), and bassoon (*BSN*). Right column: Post-synapse: Homer scaffold protein 3 (*HOMER3*), disc large MAGUK scaffold protein (*DLG*) 3 and 4, SH3 and multiple ankyrin repeat domain (*SHANK*) 3 and 1. Astrocytes: Solute carrier family 1 member (*SLC1A*) 2 and 3, S100 calcium binding protein B (*S100B*), aquaporin 4 (*AQP4*), aldehyde dehydrogenase 1 family member L1 (*ALDH1L1*), glial fibrillary acidic protein (*GFAP*). Oligodendrocytes: 2′,3′-cyclic nucleotide 3′phosphodiesterase (*CNP*), claudin 11 (*CLDN11*), myelin basic protein (*MBP*), oligodendrocyte transcription factor 2 (*Olig2*), myelin oligodendrocyte glycoprotein (*MOG*). **(B)** Representative immunohistochemical staining of iPNs by β-III tubulin and 4′,6-diamidino-2-phenylindole (DAPI) of iPNs at control condition (CTRL), following 6 h of nocodazole damage (10 µM nocodazole), following 24 h of neurite recovery in neuronal medium (CTRL-Recovery), and following 24 h of neurite recovery in neuronal medium supplemented with 100 µM PA (100 µM PA-Recovery). Scale bar 100 µm. (**C**) Fold change in neurite length following neurite recovery in the presence of 10 µM (*n* = 130), 100 µM (*n* = 119), and 1000 µM (*n* = 129) PA in comparison with CTRL-Recovery for 24 h. Data are represented as mean ± SEM, *P < 0.05, ***P < 0.001, *ns* = not significant, *n* = sum length of neurites per neuron.

Based on our previous findings, demonstrating that PA supplementation modulates neuroinflammation-associated neurodegeneration,^16,21^ we analysed the direct effect of PA on neurite regrowth in our human neuronal *in vitro* model of pwMS-specific iPNs (Fig. 1A, Supplementary Fig. 1A and B).^23^ Axonal damage was induced by treating pwMS-specific iPNs with the microtubule-destabilizing adjuvant nocodazole for 6 h, resulting in axonal beading and a significant reduction in the neurite length by 58% (*P* < 0.0001), which was followed by a significant outgrowth of the neurite length by 90% (*P* < 0.0001) after nocodazole withdrawal and recovery for 24 h (Supplementary Fig. 1B).

In the presence of 100 µM PA, neurite outgrowth was significantly increased by 26% (*P* < 0.001), compared with control recovery. Administration of 10 µM PA led to a 17% increase in the neurite length (*P* = 0.027), whereas 1000 µM PA enhanced neurite regrowth by 11% (*P* = 0.13) (Fig. 1B and C), demonstrating that PA mediates neurite recovery of pwMS-specific iPNs in a dose-dependent manner.

**Figure 2:**
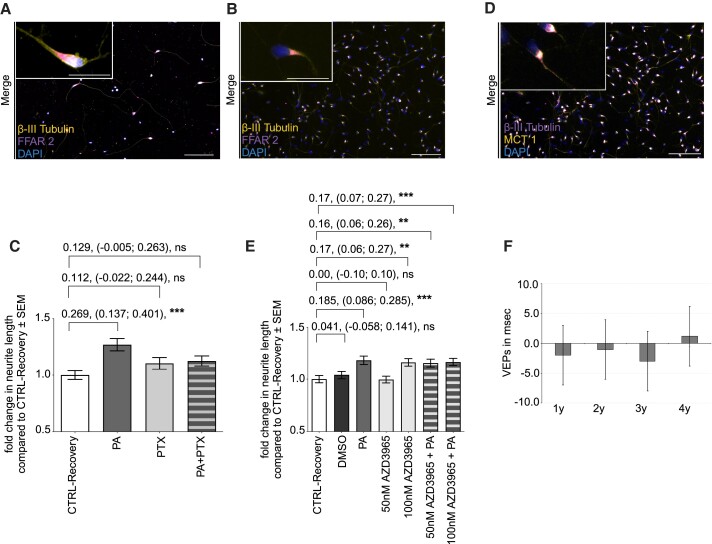
**PA promotes neuroregeneration mediated via FFAR signalling.** (**A & B**) Immunohistochemical staining of FFAR 2 and 3 expression on iPNs differentiated from pwMS. IPNs are stained positive for the neuronal marker β-III tubulin, FFAR 2 and 3, and 4′,6-diamidino-2-phenylindole (DAPI). (**C**) Treatment of iPNs with PTX in the neurite regrowth assay following neurite damage by nocodazole inhibited PA-mediated impact on neurite sum length. CTRL-Recovery (*n* = 161), 100 µM PA (*n* = 173), 250 ng PTX (*n* = 166), PA plus PTX (*n* = 161). (**D**) Immunohistochemical staining of the MCT1 expression in iPNs. Neurons are stained positive for the neuronal marker β-III tubulin, MCT1, and DAPI. Scale bar within the zoom in 20 µm, in the overview of 100 µm. (**E**) Fold change in neurite length following pre-treatment with the MCT1 inhibitor AZD3965. Control recovery (*n* = 158), DMSO (*n* = 158), PA (*n* = 159), 50 nM AZD3965 (*n* = 160), 100 nm AZD3965 (*n* = 136), 50 nm AZD3965 plus PA (*n* = 160), 100 nm AZD3965 plus PA (*n* = 160). Data are represented as mean ± SEM, **P < 0.01, ***P < 0.001, *ns* = not significant, *n* = sum length of neurites per neuron. (**F**) Stable VEPs since PA initiation. P100 latency (in ms) did not change significantly. *N* = 35, *n* = 67, first year (*n* = 60), second year (*n* = 34), third year (*n* = 18), fourth year (*n* = 18), *N* = individuals analysed, *n* = eyes analysed. Analysed by Wilcoxon–Mann–Whitney test.

To investigate an involvement of FFAR 2 and 3 signalling, first we confirmed receptor expression on pwMS-specific iPNs via immunohistochemistry (Fig. 2A and B). Afterwards, PTX, as a specific antagonist for FFAR 2 and 3 downstream signalling of the Gα subunit Gα_i/o_, was administered prior to PA treatment. Inhibition of FFAR 2 and 3 downstream signalling resulted in an inhibition of PA impact on increased neurite outgrowth in iPNs (Fig. 2C). Administration of the specific MCT1 inhibitor AZD3965 did not block PA-dependent neurite growth. Therefore, we conclude that the observed increase in neurite growth by PA is rather mediated by an activation of the intrinsic pathway of the Gα_i/o_ subunit of FFARs than by intracellular transport via the MCT 1 (Fig. 2D and E).

### Supplementation of propionic acid favours stabilization of visually evoked potentials

In multiple sclerosis, decelerated neuronal transmission between the optic nerves and the visual cortex is dependent on demyelination and axonal damage. Hence, we examined visually evoked potentials (VEPs) in people with autoimmune, demyelinating disorders who were supplemented with PA over a time period of 4 years. Evaluation of transmission rates revealed a stabilization of VEPs mediated by PA supplementation: In comparison to the baseline P100 latency (124.2 ± 16.1 ms), after the first year of PA intake, VEPs are reduced by −2.0 ms (*P* = 0.343), the second year by −1.1 ms (*P* = 0.212), and the third year by −3.0 ms (*P* = 0.274), and in the fourth year, VEP latencies are increased by 1.2 ms (*P* = 0.224) (Fig. 2F). Although we did not observe a significant reduction in VEPs, our data imply that PA supplementation possibly promotes stabilization of ongoing neurodegeneration in demyelinating disorders, emphasizing the clinical relevance on neuroregeneration mediated by PA.

### Proteome analyses revealed alterations in protein groups of induced primary neurons recovered in the presence of propionic acid

**Figure 3:**
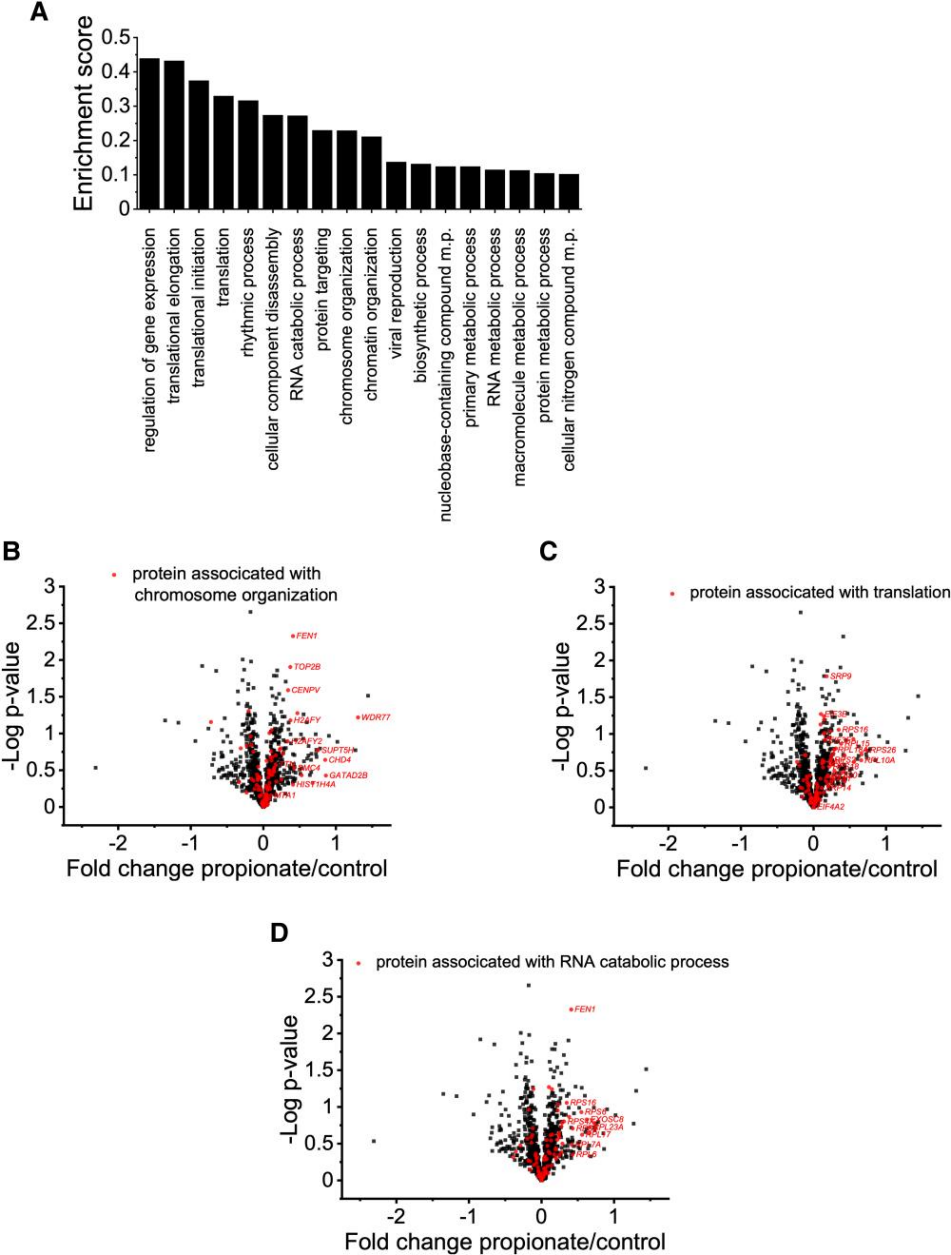
**Proteomic analyses of pwMS-specific iPNs recovered in the presence of PA.** (**A**) Enrichment score of altered protein expression in iPNs following recovery in the presence of 100 µM PA (*n* = 5). For statistical and categorical analysis, normalized label-free quantification intensities were log2-transformed and missing values imputed with values from a downshifted (1.8 standard deviations) normal distribution (width: 0.3 standard deviations). Difference of group means was calculated and used for one-dimensional annotation enrichment analysis based on Wilcoxon–Mann–Whitney tests. Given *q*-values have been calculated from *P*-values by the method of Benjamini and Hochberg. Proteins associated with chromosome organization (**B**), translation (**C**), and RNA catabolic processes (**D**) are highlighted in volcano plot. The provided fold change in **B**–**D** represents the difference of the group mean values of the log2-transformed label-free quantification intensities.

**Figure 4:**
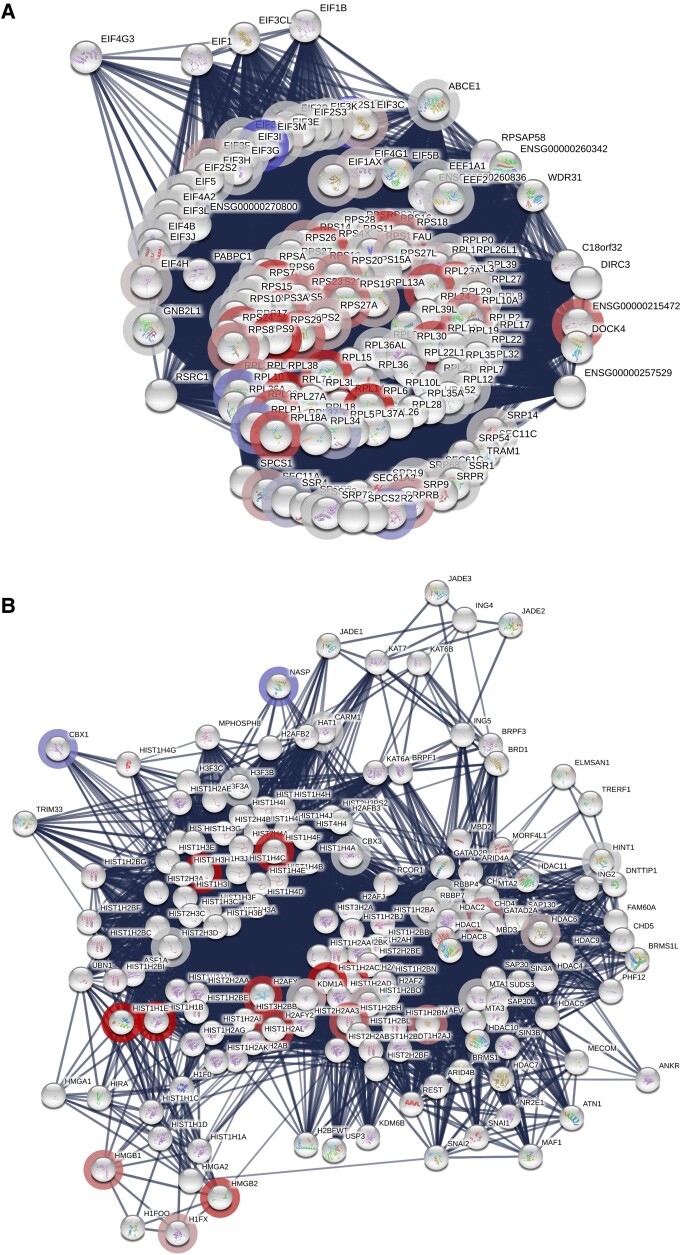
**STRING analyses of pwMS-specific iPNs recovered in the presence of PA.** (**A**) STRING analysis of protein interactions associated with GTP hydrolysis and joining of the 60S ribosomal subunit and protein export pathway. (**B**) STRING analysis of protein interactions associated with nucleosome and histone deacetylase complex. Proteins showing higher abundances upon PA treatment are marked by red halos and proteins showing lower abundances by blue halos.

Quantitative mass spectrometry of iPNs, recovered in the presence of 100 µM PA for 24 h in comparison to control-recovered iPNs following previous damage by nocodazole, was performed. On functional but not individual protein level, changes in the abundance of functionally related proteins could be observed, particularly in protein groups involved in metabolism, ribosomal and translational processes, and chromatin assembly and associated histone deacetylase complexes, by means of one-dimensional annotation enrichment of Gene Ontology (GO) terms^29^ (Fig. 3A–D) (Table 2). For chromosome organization and RNA catabolic processes, ribosomal proteins (RPLs) showed higher abundances following recovery of PA-treated iPNs. Network analysis by STRING revealed protein interactions associated with guanine triphosphate (GTP) hydrolysis, and joining of the 60S ribosomal subunit and protein export pathway exhibited an altered protein abundance due to PA recovery. Associated proteins included RPLs as well as elongation initiation factors (EIFs), proteins related to translation and protein expression (Fig. 4A). The altered abundance of proteins within the nucleosome and histone deacetylase complex network revealed an involvement of chromatin assembly. Thereby, histone cluster proteins (i.e. HIST1H4I or HIST3H2A) display core components of the nucleosome, which are important for DNA packing and for chromatin assembly (Fig. 4B). Our analyses suggest that PA directly targets cellular chromatin assembly to facilitate translational processes and protein expression, thereby orchestrating neurite recovery.

**Table 2:** One-dimensional annotation enrichment of processes in iPNs treated with PA

Type	Name	Size	Score	*P*-value	Benj. Hoch. FDR
GOMF	Structural constituent of ribosome	52	0.54	4.0E^−11^	6.3E^−08^
GOCC	Ribosome	50	0.53	1.7E^−10^	1.4E^−07^
GOBP	Nuclear-transcribed mRNA catabolic process, nonsense-mediated decay	65	0.46	2.4E^−10^	1.3E^−06^
KEGG	Ribosome	52	0.51	2.7E^−10^	6.3E^−08^
GOBP	Translational termination	55	0.49	6.0E^−10^	1.7E^−06^
GOBP	Translational elongation	59	0.47	1.2E^−09^	2.2E^−06^
GOBP	Translational initiation	83	0.38	7.6E^−09^	1.1E^−05^
GOBP	SRP-dependent cotranslational protein targeting to membrane	66	0.42	8.0E^−09^	8.8E^−06^
GOBP	Cotranslational protein targeting to membrane	66	0.42	8.0E^−09^	7.4E^−06^
GOCC	Nucleosome	15	0.85	1.5E^−08^	6.2E^−06^
GOBP	Viral transcription	67	0.41	1.6E^−08^	1.3E^−05^
GOBP	Protein targeting to ER	67	0.41	1.8E^−08^	1.1E^−05^
GOBP	Establishment of protein localization in endoplasmic reticulum	67	0.41	1.8E^−08^	1.2E^−05^
GOBP	Establishment of protein localization to organelle	90	0.35	2.2E^−08^	1.2E^−05^
GOBP	Nuclear-transcribed mRNA catabolic process	85	0.36	2.9E^−08^	1.4E^−05^
GOBP	Protein complex disassembly	65	0.40	4.1E^−08^	1.9E^−05^
GOBP	Translation	107	0.32	4.4E^−08^	1.9E^−05^
GOBP	RNA catabolic process	94	0.34	5.1E^−08^	2.0E^−05^
GOBP	Viral infectious cycle	78	0.36	6.3E^−08^	2.3E^−05^
GOBP	Cellular protein complex disassembly	64	0.40	8.1E^−08^	2.8E^−05^
GOBP	mRNA catabolic process	88	0.34	8.7E^−08^	2.8E^−05^
GOBP	Cellular component disassembly	119	0.29	9.9E^−08^	3.0E^−05^
GOBP	Macromolecular complex disassembly	78	0.35	1.4E^−07^	4.1E^−05^
GOBP	Cellular component disassembly at cellular level	118	0.29	1.7E^−07^	4.7E^−05^
GOBP	Cellular process involved in reproduction	108	0.30	2.5E^−07^	6.6E^−05^
GOBP	Cellular macromolecular complex disassembly	77	0.35	2.7E^−07^	6.6E^−05^
GOBP	Reproductive process	185	0.23	3.4E^−07^	8.2E^−05^
GOBP	Protein targeting to membrane	73	0.34	6.8E^−07^	1.6E^−04^
GOCC	Protein–DNA complex	19	0.66	8.2E^−07^	2.2E^−04^
GOCC	Cytosolic small ribosomal subunit	29	0.53	1.0E^−06^	2.1E^−04^
GOBP	Cellular macromolecular complex subunit organization	214	0.20	1.8E^−06^	3.9^E−04^
GOCC	Small ribosomal subunit	30	0.50	2.5E^−06^	4.1E^−04^
GOMF	Nucleic acid binding	411	0.15	1.2E^−05^	9.2E^−03^
GOBP	Viral reproductive process	101	0.26	1.6E^−05^	3.4E^−03^
GOBP	Macromolecular complex subunit organization	273	0.17	1.7E^−05^	3.4E^−03^
GOCC	Cytosolic large ribosomal subunit	25	0.50	1.8E^−05^	2.5E^−03^
GOCC	Large ribosomal subunit	25	0.50	1.8E^−05^	2.1E^−03^
GOBP	mRNA metabolic process	232	0.17	3.1E^−05^	6.1E^−03^
GOBP	Gene expression	327	0.15	5.0E^−05^	9.5E^−03^
GOBP	Macromolecule biosynthetic process	299	0.14	1.1E^−04^	2.0E^−02^
GOBP	Macromolecule catabolic process	191	0.17	1.2E^−04^	2.1E^−02^
GOBP	Cellular macromolecule biosynthetic process	296	0.14	1.4E^−04^	2.4E^−02^
GOBP	Cellular macromolecule catabolic process	180	0.17	1.5E^−04^	2.6E^−02^
GOBP	Protein complex subunit organization	175	0.17	2.3E^−04^	3.7E^−02^
GOBP	Chromosome organization	111	0.21	2.8E^−04^	4.5E^−02^
GOCC	Intracellular non-membrane-bounded organelle	430	0.12	4.6E^−04^	4.2E^−02^
GOCC	Non-membrane-bounded organelle	430	0.12	4.6E^−04^	4.7E^−02^

GOBP, Gene Ontology biological processes; GOCC, Gene Ontology cellular components; GOMF, Gene Ontology molecular function; KEGG, Kyoto Encyclopedia of Genes and Genomes.

### Neuroregeneration by propionic acid is mediated via histone deacetylase inhibition and the antioxidant stress response

**Figure 5:**
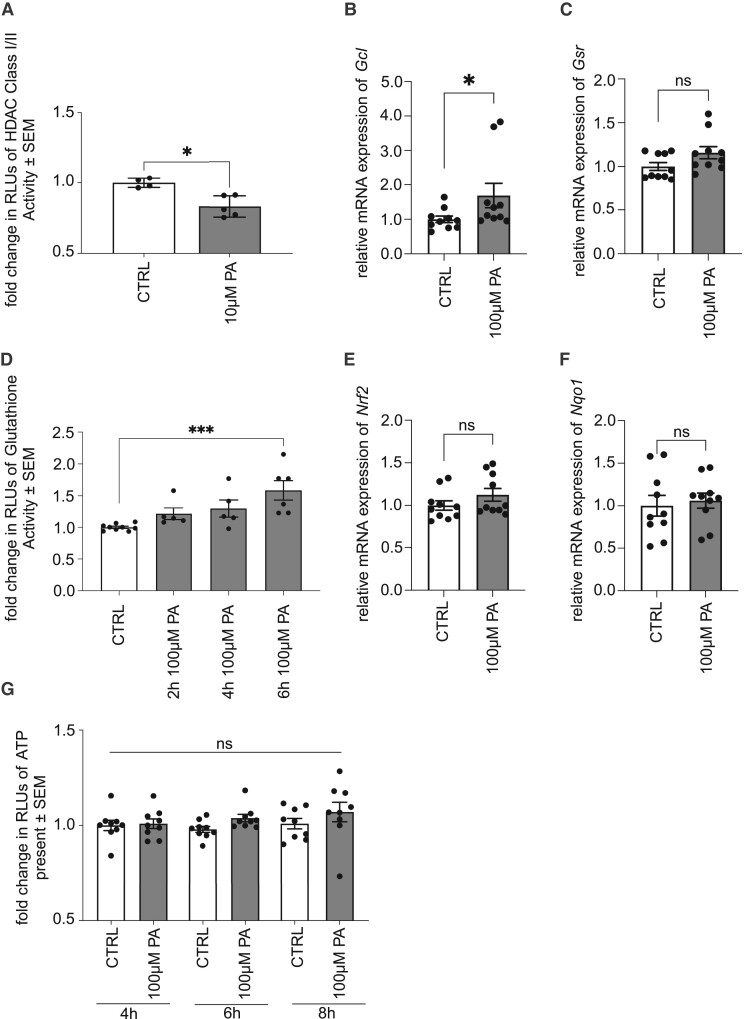
**Increased neurite growth of pwMS-specific iPNs by PA is mediated by inhibition of class I/II HDAC and activation of the anti-oxidative response.** (**A**) HDAC class I/II activity was inhibited by treatment of pwMS iPNs with PA after 30 min (CTRL *n* = 4, 100 µM PA *n* = 5). (**B**) Fold change of relative mRNA expression of GCL was significantly increased following recovery in the presence of PA after 6 h (*n* = 10). (**C**) mRNA expression of GSR was tendentially increased (*n* = 10). (**D**) Glutathione activity was increased following cultivation of pwMS-specific iPNs in the presence of 100 µM PA after 6 h (CTRL *n* = 9; 2 h 100 µM PA *n* = 5; 4 h 100 µM PA *n* = 5; 6 h 100 µM PA *n* = 6). (**E**) No difference was observed in mRNA expression of *Nrf2* (*n* = 10) (**F**) and *Nqo1* after 6 h of recovery (*n* = 10). (**G**) Luminescent analysis of iPN’s ATP production during cultivation in the presence of 100 µM PA for 4, 6, and 8 h (*n* = 9). Data are represented as mean ± SEM and analysed by the Mann–Whitney test (**A**–**C**; **E**; **F**) and by the Kruskal–Wallis test with Dunn’s multiple comparison (**D**; **G**), *P < 0.05, ***P < 0.001, *ns* = not significant.

Since proteome analysis revealed alterations in the chromatin assembly and an influence on subsequent protein biosynthesis, we further analysed the impact of PA treatment of iPNs in the context of the inhibition of HDAC class I/II activity via luminescence analysis, as a known target of FFAR downstream signalling. We identified that cultivation of pwMS-specific iPNs in the presence of 100 µM PA leads to a significant reduction of HDAC class I/II activity after 30 min of treatment (*P* = 0.0159) (Fig. 5A). Alterations in the expression levels of histone H3 acetylation of lysine 9 (H3K9) as well as of HDACs 1, 5, and 6, which are known to be involved in neurodegeneration, could not be detected by western blot analysis after 24 h (Supplementary Figs. 2A–D, 3A–D, 5, and 6), leading to the assumption that modifications in histone acetylation are induced immediately following PA-mediated FFAR activation.

Via PCR analysis, we identified that administration of PA significantly increased the mRNA expression of the glutamate–cysteine ligase (GCL) after 6 h (*P* = 0.0336) (Fig. 5B), which is part of the anti-oxidative response of the glutathione pathway. Furthermore, mRNA levels of the glutathione reductase (GSR) were tendentially increased when iPNs were recovered in the presence of PA for 6 h (*P* = 0.0843) (Fig. 5C). Hence, GSR activation was further investigated by luminescent analysis. We identified a significant increase in GSR activity following cultivation of pwMS-specific iPNs for 6 h (*P* = 0.0073) (Fig. 5D). However, this effect was not mediated by activation of the nuclear factor erythroid 2-related factor 2 (*Nrf2*), or *Nrf2* downstream target NAD(P)H quinone oxidoreductase 1 (*Nqo1*) (Fig. 5E and F). Analysis regarding the metabolic activity following cultivation of iPNs in the presence of 100 µM PA revealed no increase in neuronal ATP production within 4–6 h (Fig. 5G).

### Neurite recovery of people with multiple sclerosis-specific induced primary neurons is enhanced by administration of butyric acid

**Figure 6:**
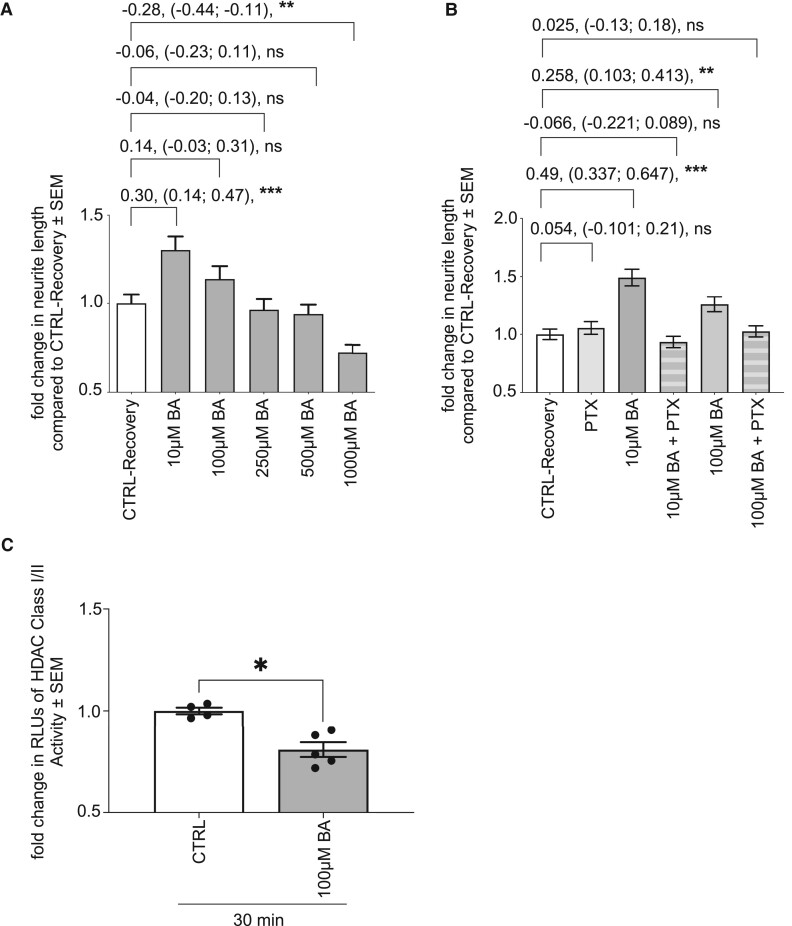
**Butyric acid increases neuroregeneration in pwMS-specific iPNs.** (**A**) Neurite recovery assay of iPNs in the presence of BA. CTRL-Recovery (*n* = 129), 10 µM (*n* = 120), 100 µM (*n* = 120), 250 µM (*n* = 120), 500 µM (*n* = 120), 1000 µM (*n* = 120). (**B**) BA-mediated impact on neurite regrowth by nocodazole was inhibited by PTX treatment. CTRL-Recovery (*n* = 120), PTX (*n* = 120), 10 µM BA (*n* = 120), 10 µM BA plus PTX (*n* = 120), 100 µM BA (*n* = 120), 100 µM BA plus PTX (*n* = 120). Data are represented as mean ± SEM, *n* = sum length of neurites per neuron. (**C**) HDAC class I/II activity was inhibited by treatment of pwMS iPNs with BA after 30 min (CTRL *n* = 4; 100 µM BA *n* = 5). Data are represented as mean ± SEM, analysed by the Mann–Whitney test (**C**), *P < 0.05, **P < 0.01, ***P < 0.001, *ns* = not significant.

**Figure 7:**
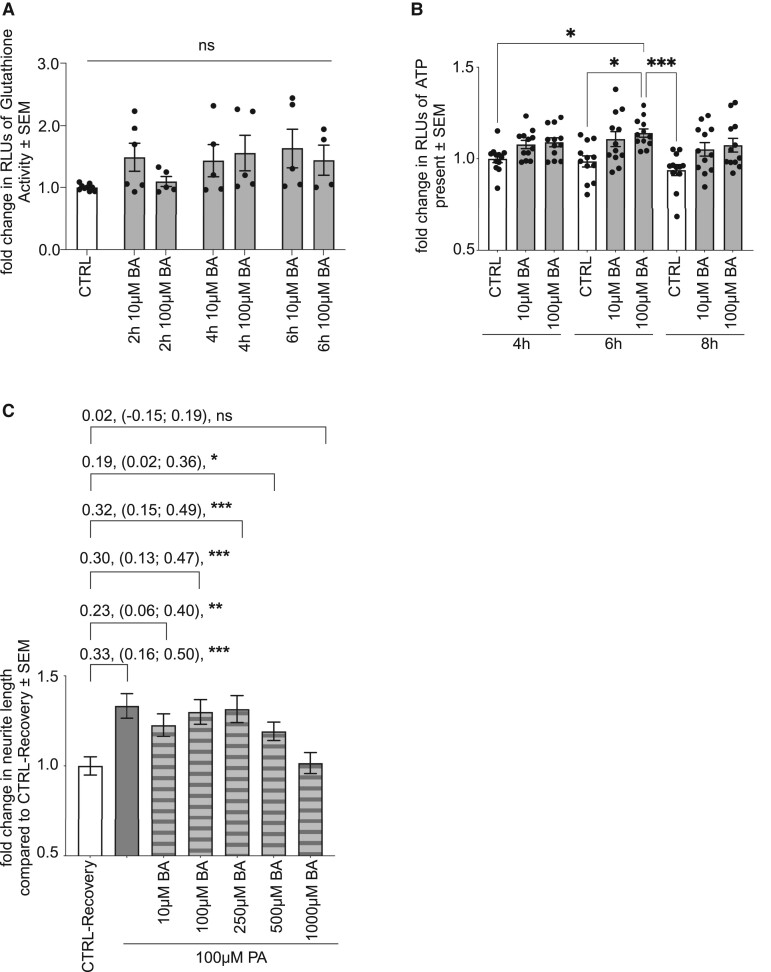
**BA’s neuroregenerative impact on iPNs is enhanced by administration of PA and increases iPN metabolic activity.** (**A**) GSR activity in iPNs following treatment with BA for 6 h displayed no alterations evaluated by luminescent analysis (CTRL *n* = 9; 2 h 10 µM BA *n* = 6; 2 h 100 µMA BA *n* = 5; 4 h 10 µM BA *n* = 5; 4 h 100 µM BA *n* = 5; 6 h 10 µM BA *n* = 5; 6 h 100 µM BA *n* = 4). (**B**) Luminescent analysis of iPN’s ATP production during cultivation in the presence of 10 µM and 100 µM BA for 4, 6, and 8 h (*n* = 12). Data are represented as mean ± SEM, analysed by the Kruskal–Wallis test with Dunn’s multiple comparison. (**C**) Neurite recovery assay by combined treatment of different BA concentrations and 100 µM PA. CTRL-Recovery (*n* = 120), 10 µM BA (*n* = 120), 100 µM BA (*n* = 120), 250 µM BA (*n* = 120), 500 µM BA (*n* = 120). Data are represented as mean ± SEM, *n* = sum length of neurites per neuron, *P < 0.05, **P < 0.01, ***P < 0.001, *ns* = not significant.

We further investigated the impact of the SCFA BA, as another metabolite discussed to play a role in neuromodulatory processes. Accordingly, neurite recovery assays in the presence of different concentrations of BA were performed. IPN recovery for 24 h in the presence of 10 µM BA resulted in an enhanced neurite outgrowth, displayed by a significant increase by 30% (*P* < 0.001) (Fig. 6A). Since other BA concentrations did not alter neurite lengths, administration of 1000 µM BA resulted in significantly shortened neurites of 72% (*P* = 0.001). Equally, PTX administration inhibited BA-mediated influence on neurite recovery in iPNs (Fig. 6B). Therefore, these results exhibited that BA distinctly modified neurite recovery and also mediated via FFAR signalling. BA administration also inhibited HDAC class I/II activity after 30 min of treatment evaluated by luminescent assay (*P* = 0.0159) (Fig. 6C). However, there was no increase in GSR activity following treatment of pwMS-specific iPNs with 10 µM or 100 µM BA (Fig. 7A). Instead, administration of 100 µM BA resulted in a significant increase in ATP production of pwMS-specific iPNs, most pronounced after 6 h of treatment of approximately 14% (*P* = 0.0298) (Fig. 7B).

### Improved neuroregeneration in induced primary neurons mediated by propionic acid is promoted by administration of butyric acid

Additionally, neurite recovery assays were conducted in combined treatment of PA and BA on iPNs, by supplementation of 100 µM PA to different BA concentrations during recovery from nocodazole damage for 24 h. The combined treatment with different BA concentrations together with 100 µM PA improved the neuroregeneration by PA, displayed by a significant elongation of the sum length of neurites around 23% in the presence of 10 µM BA (*P* = 0.009), 30% for 100 µM BA (*P* < 0.001), 32% for 250 µM BA (*P* < 0.001), and 19% for 500 µM BA (*P* = 0.0026). Additionally, administration of PA to the 1000 µM BA condition abrogated the inhibition of neuroregeneration by BA (Fig. 7C). Therefore, we conclude that the neuroregenerative impact of PA is further improved by administration of BA. Since BA exerts greater influence on ATP production of iPNs compared with PA, we conclude that the underlying mode of action of PA on neuroregeneration is rather based on an increased protein expression, promoting neurite outgrowth by activation of the anti-oxidative response, whereas BA enhances cellular metabolism in iPNs.

### Neuroprotective effects of propionic acid and butyric acid are not mediated by inhibition of neuronal apoptosis

Following cultivation of pwMS-specific iPNs in the presence of different PA concentrations, different BA concentrations as well as in the combined treatment displayed no impact on neuronal activation of caspases 3 and 7 (Supplementary Fig. 4A and B). Further, neither therapeutic nor prophylactic treatment with SCFAs was able to prevent apoptosis in iPNs, which was induced by administration of the protein kinase inhibitor staurosporine (Supplementary Fig. 4C and D). Therefore, we conclude that neuroregenerative properties of SCFA treatment are mediated by directly targeting the regenerative capacities of neurons rather than by preventing apoptosis in iPNs.

## Discussion

In this study, we demonstrated that the SCFAs PA and BA promote neuroregeneration in an *in vitro* model of pwMS-specific iPNs by independent mechanisms. We were able to show that the neuroprotective effects of PA and BA were mediated by the FFAR signalling pathway, downstream leading to inhibition of class I/II HDAC activity. Especially for PA, proteome analysis revealed an influence on metabolic and translational processes, associated with alteration in the chromatin assembly, the latter supported by our findings of posttranslational modifications mediated by PA treatment. Improvement of neurite regrowth by PA was mediated by enhancing the anti-oxidative response via activation of the glutathione pathway, whereas administration of BA increases neuronal ATP synthesis. Furthermore, by combined treatment of pwMS iPNs with BA and PA, we were able to demonstrate that BA promotes the neuroregenerative properties of PA. Additionally, observed effects were not mediated by modulation of anti-apoptotic mechanisms. Our previous *in vivo* data highlighting the neuroprotective effects of PA supplementation^16^ were further supported clinically by evaluation of VEPs in people with demyelinating diseases, indicating an attenuation in demyelination and axonal loss. However, VEP analyses were only indicative for a stabilization in disease progression and needed to be further evaluated.

Beyond their immunomodulatory attributes,^16,32,33^ SCFAs are highly debated to exert modulatory effects on neurons and glial cells. We previously confirmed neuroprotective effects of PA and β-hydroxybutyrate on cells of the PNS, namely on dorsal root ganglia (DRGs) and Schwann cells. Accordingly, neuroprotective effects were mediated by FFAR receptor signalling, inhibition of class I/II HDACs, and activation of the anti-oxidative response.^34^ In the CNS, SCFAs have been shown to enhance remyelination by targeting oligodendrocyte function^35^ and to modulate microglial homeostasis.^36^ Additionally, SCFAs were shown to protect BBB integrity by modulation of tight junctions.^37^ Especially PA was demonstrated to protect the BBB from oxidative stress.^38^ Although administration of BA exerted no significant impact on the anti-oxidative response in pwMS-specific iPNs, we observed an enhancement of cellular metabolism in iPNs by BA. As a primary energy source of colonocytes, BA can be metabolized within mitochondria via the TCA cycle.^39,40^ Accordingly, SCFAs comprise important energy sources and are assumed to be involved in the communication within the gut and enteric neurons.^41^ Based on our findings, we hypothesize that the increase in neurite regrowth in iPNs caused by BA is based on an improvement of mitochondrial activity. Axonal degeneration leads to an impairment of organelle transport,^42^ including mitochondria, which are essential to provide for the energy supply required for neurons’ capability to regenerate or sustain neuroinflammation.^43^ In multiple sclerosis, axonal transport deficits mainly drive neurodegeneration and are induced by ROS, among others. Besides diminished transport of mitochondria to lesion sites, ROS also causes mitochondrial death, thereby enhancing neurodegenerative processes.^3^ Therefore, strengthening of the anti-oxidative response can improve axonal transport, thereby increasing neuroregeneration.^42^ Additionally, in regenerating axons, an increase in mitochondrial density supports local energy supply that is crucial to promote growth cone expansion and neurite regeneration.^43,44^ Identification of compensatory approaches to counteract deficits in energy supply and disruption of the cytoskeletal integrity of axons comprise a high clinical implication.^43,45^ An enhancement or restoration of mitochondrial activity by BA might therefore facilitate neuroregeneration in iPNs.

Via immunohistochemistry, expression of the SCFA-specific FFAR 2 and 3 was confirmed in pwMS-specific iPNs. Since administration of the FFAR 2- and 3-specific antagonist PTX^46^ inhibited the neuroregenerative effects mediated by PA and BA, our data imply that FFAR 2 and 3 downstream signalling mediates the neuroprotective effects of SCFA administration. FFARs’ heterotrimeric G-protein consists of Gα, β, and γ subunit, intrinsically leading to histone acetylation and inhibition of HDAC activity.^33^ Administration of PTX downstream antagonizes specifically the activation of the FFA receptor subunit Gα subclass Gαi/o.^46^ As inhibition of the MCT1 did not reduce neurite regrowth caused by PA, we therefore conclude that the impact of SCFAs on iPNs is mainly mediated by FFAR signalling.

Besides FFAR signalling, SCFAs regulate chromatin modifications and subsequent protein expression.^47^ HDACs are involved in the regulation of axonal degeneration,^48^ as well as synaptic plasticity.^49^ Hence, active posttranslational modifications are already examined as therapeutic target for the treatment of neurological disorders.^50,51^ We analysed the protein expression of HDACs 1, 5, and 6, known to be involved in neurodegenerative processes.^48,52,53^ Although we were not able to detect an altered expression of HDCAs 1,5, and 6 after 24 h of recovery, we observed a global reduction in HDAC class I/II activity following SCFA treatment already after 30 min, therefore concluding that modulation of HDACs is initiated immediately following FFAR activation.

In multiple sclerosis pathogenesis, the prevalent dysbiosis of the commensal gut microbiome, particularly reflected by a decreased abundance of SCFA-producing bacterial strains,^19,20^ is hypothesized as a causal link for the deficiency of systemic SCFA availability in pwMS.^16-18^ This hypothesis is supported by the assumption that the so-called western diet lacks important amount of fibres, leading to a deficiency in systemically available SCFA.^54^ Various studies point to an involvement of the lack of a fibre-rich diet and the accumulation of non-communicable diseases.^55^ The impact of microbial metabolites on CNS physiology is highly debated, and our understanding concerning the mechanism of the microbiome–gut–brain axis remains largely scarce. Recently, the implication of gut microbiome alterations in diseased states of the CNS^56-58^ and even in behavioural changes has gained increasing attention.^22,59,60^ Commensal microbiota were shown to be involved in defining brain functions as mood,^61^ stress,^62^ and anxiety.^63-65^ Especially the absence of microbial metabolites during the embryonic stage considerably afflicts brain development and physiology,^66-68^ indicating a substantial role of metabolites produced by the commensals in CNS homeostasis. Due to the prevalent symbiosis of the commensal microbiota with the host’s immune system,^69^ modifying immunomodulatory properties by dietary intervention comprises a promising therapeutic target to counteract neuroinflammation and accumulating neurodegeneration.^22^

In the present study, we demonstrated a possible direct neuroprotective effect of SCFA—especially of PA—on the regenerative capacity of neurons of the CNS. Based on our previous findings, evaluating PA as distinct modulator for Treg differentiation and functionality,^16,21^ we were now able to show that PA alleviates neuroinflammation in addition to its immunomodulatory attributes within a reverse-translational *disease-in-a-dish* model. Both PA and BA significantly enhanced neurite recovery, mediated by FFAR signalling and downstream inhibition of the activity of HDAC class I/II. Assuming that BA is a potentiator of PA’s neuroprotective properties in pwMS-specific iPNs, we identified that neuroregeneration by PA is rather mediated by activation of the anti-oxidative response in iPNs mediated via the glutathione pathway, whereas BA increases neuronal metabolism by an enhanced ATP synthesis.

To our knowledge, this study is the first to show a direct neuroregenerative impact of SCFA on human neurons. However, currently our model is limited to iPNS from three donors, possibly driving the likelihood of heterogeneity between experiments conducted with pwMS-specific iPNs. Co-factors including genetic heterogeneity between individuals and sex differences are discussed to drive variability between respective cell lines.^70-72^ To account for possible cell donor effects within the neurite recovery assays, we included a corresponding random effect to our regression models.

However, while we are confident in our results, due to the experimental setup and limited number of cell donors, we are not able to definitively rule out further possible unobserved random effects beyond the cell donor effect. We performed sensitivity analyses where we tested the effects on neurite length using cluster-robust CR2 SE^73^ in addition to the random donor effect, providing a more conservative point of view regarding confidence intervals and corresponding *P*-values (Supplementary Table 1).

Furthermore, reprogramming might influence the epigenetic background.^74^ Therefore, further studies using larger numbers of donors for the differentiation of pwMS-specific iPNs will help to address MS heterogeneity in our model as well as different disease forms.

However, we assume that our model reflects clinical aspects of PA supplementation and physiological processes. Since progressive forms of multiple sclerosis are almost lacking suitable treatment options, administration of PA might decelerate ongoing neuronal loss, by attenuating neuroinflammation and retarding the accumulation of neurodegeneration. Consequently, as add-on to existing DMTs for multiple sclerosis, SCFAs might support a delayed disease progression.^75,76^ This hypothesis is supported by the beneficial outcome of dietary intervention in the context of multiple sclerosis.^77^ In neurodegenerative diseases as multiple sclerosis, the synergistic effect of SCFA on immunomodulation, neuroinflammation, and neuronal regeneration might provide a suitable treatment option for pwMS.

## Supplementary Material

fcae182_Supplementary_Data

## Data Availability

All data of this study are included in the paper and available from the corresponding author, upon request. The mass spectrometry proteomics data have been deposited to the ProteomeXchange Consortium via the Proteomics Identifications (PRIDE) Database^31^ partner repository with the dataset identifier PXD040184.
