# Supplementary material for: Propionic acid promotes neurite recovery in damaged multiple sclerosis neurons
Source: Brain Commun. 2024 Jun 3;6(3):fcae182. doi: 10.1093/braincomms/fcae182 (PMC11184351; doi:10.1093/braincomms/fcae182)
Supplement: fcae182_Supplementary_Data [file fcae182_supplementary_data.pdf]

Supplementary material

Supplementary Figures

Supplementary Figure 1

A

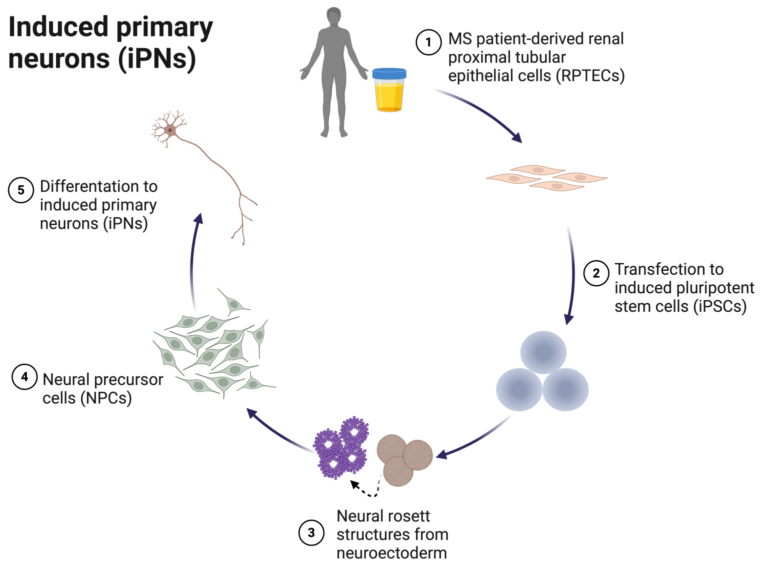

B

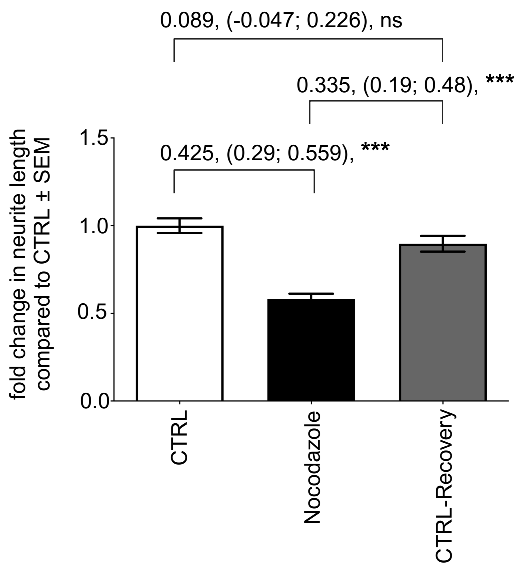

Supplementary Figure 1. Model of pwMS-specific iPNs and neurite recovery assay. (**A**) Depictive representation of the generation of induced primary neurons (iPNs) from pwMS renal proximal tubular epithelial cells (RPTECs) (1). RPTECs are reprogrammed to induced pluripotent stem cells (iPSCs) by transfection via electroporation with episomal plasmids (2). Embryoid bodies (EB) are generated from iPSC colonies. By inhibition of the development of meso- and entoderm, neuroectoderm develops from ectoderm by cultivation in neural stem cell medium, leading to the formation of neural rosette structures (3). Neural precursor cells (NPCs) are isolated from neural rosette structures (4) and differentiated into iPNs by neuronal differentiation medium (5). (**B**) Quantification of the sum length of neurite length following nocodazole treatment ($N = 3$, CTRL $n = 142$, Nocodazole $n = 94$, CTRL-Recovery $n = 90$). Data are represented as mean ± SEM, ***p < 0.001, ns = not significant, n = sum length of neurites per neuron.

Supplementary Figure 2

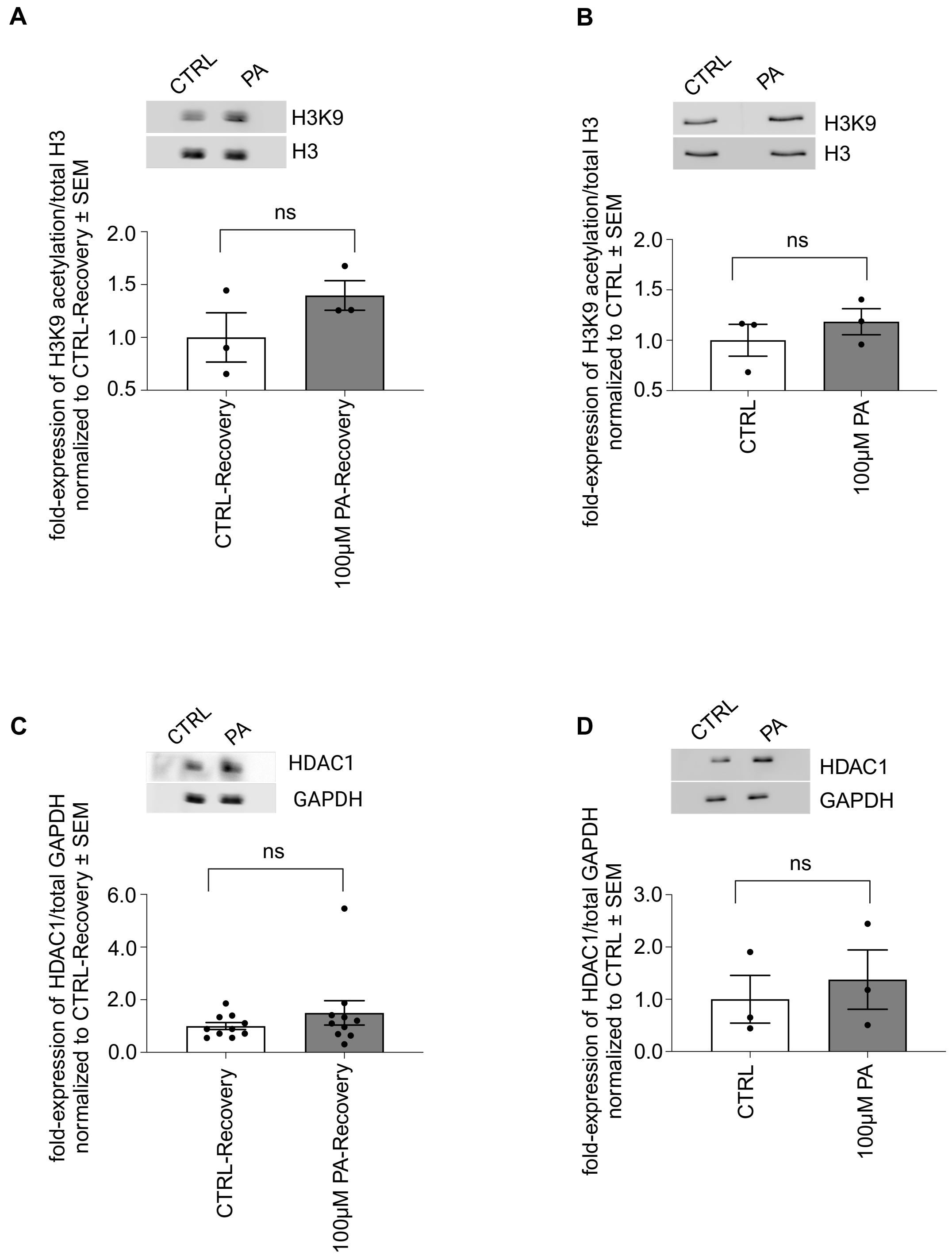

Supplementary Figure 2. Western Blot analyses of histone acetylation and HDAC 1 activity. (**A**) Example western blot analysis of histone H3 acetylation at lysine 9 (K9) in iPNs following recovery for 24 h from neurite damage by nocodazole in the presence of 100 µM PA compared to control-recovery ($n = 3$). (**B**) Example western blot analysis of histone H3

acetylation of lysine 9 (K9) in iPNs, which were cultivated in the presence of 100 µM PA for 24 h without previous damage (*n* = 3). (**C**) Example western blot analysis of histone deacetylase (HDAC) 1 expression in iPNs following recovery for 24 h from neurite damage by nocodazole in the presence of 100 µM PA compared to control-recovery (*n* = 10). (**D**) Example western blot analysis of histone deacetylase (HDAC)1 expression in iPNs, which were cultivated in the presence of 100 µM PA for 24 h without previous damage (*n* = 3). Data are represented as mean ± SEM, and analyzed by Mann Whitney-Test, ns = not significant.

Supplementary Figure 3

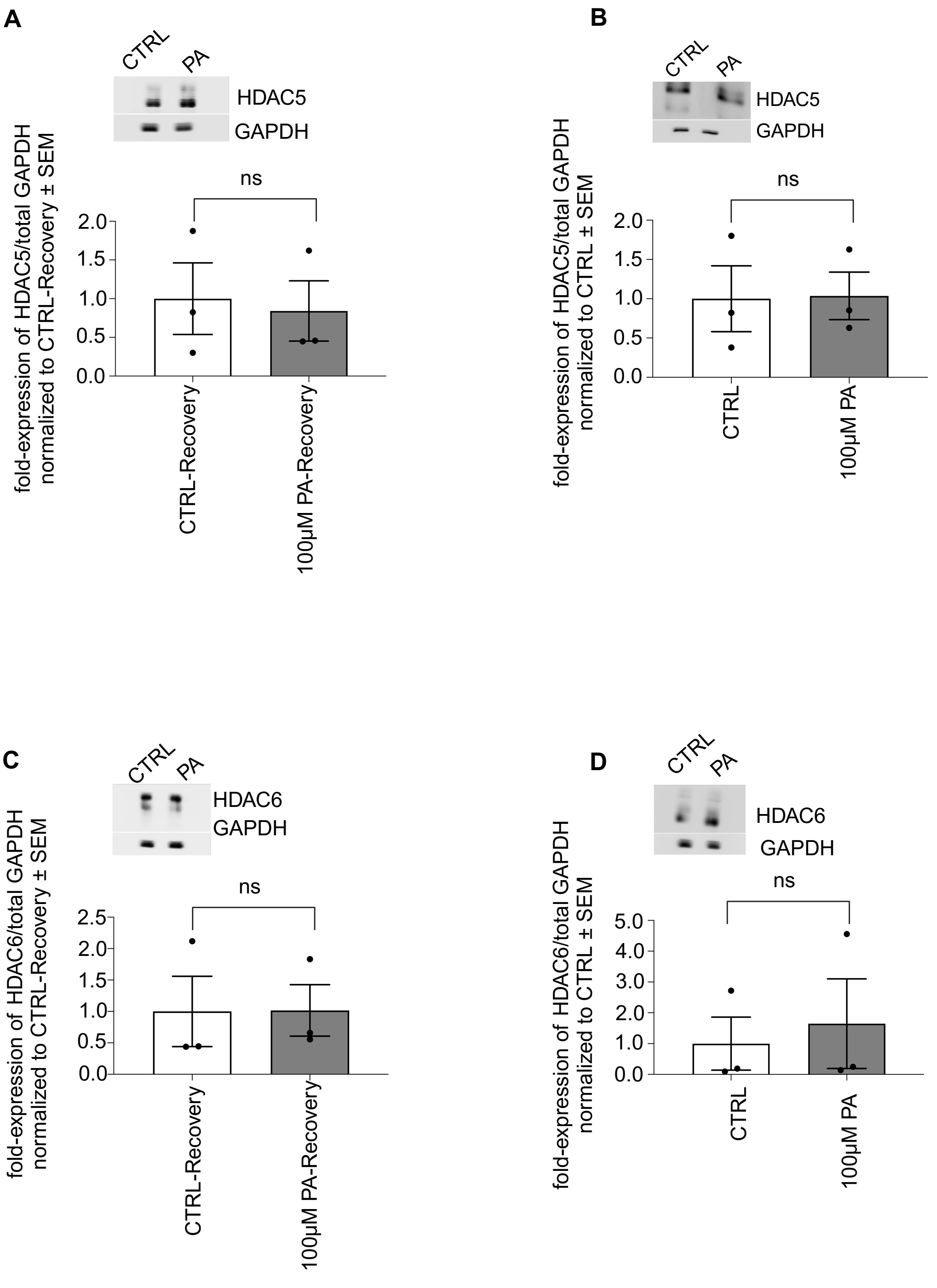

Supplementary Figure 3. Western Blot analyses of HDAC 5 & 6 activity. (**A**) Example western blot analysis of histone deacetylase (HDAC) 5 expression in iPNs following recovery for 24 h from neurite damage by nocodazole in the presence of 100 µM PA compared to control-recovery (*n* = 3). (**B**) Example western blot analysis of histone deacetylase (HDAC) 5

expression in iPNs without previous damage cultivated in the presence of 100 µM PA for 24 h compared to control condition (*n* = 3). (**C**) Example western blot analysis of histone deacetylase (HDAC) 6 expression in iPNs following recovery for 24 h from neurite damage by nocodazole in the presence of 100 µM PA compared to control-recovery (*n* = 3). (**D**) Example western blot analysis of HDAC 6 expression in iPNs, which were cultivated in the presence of 100 µM PA for 24 h without previous damage (*n* = 3). Data are represented as mean ± SEM, and analyzed by Mann Whitney-Test, ns = not significant.

Supplementary Figure 4

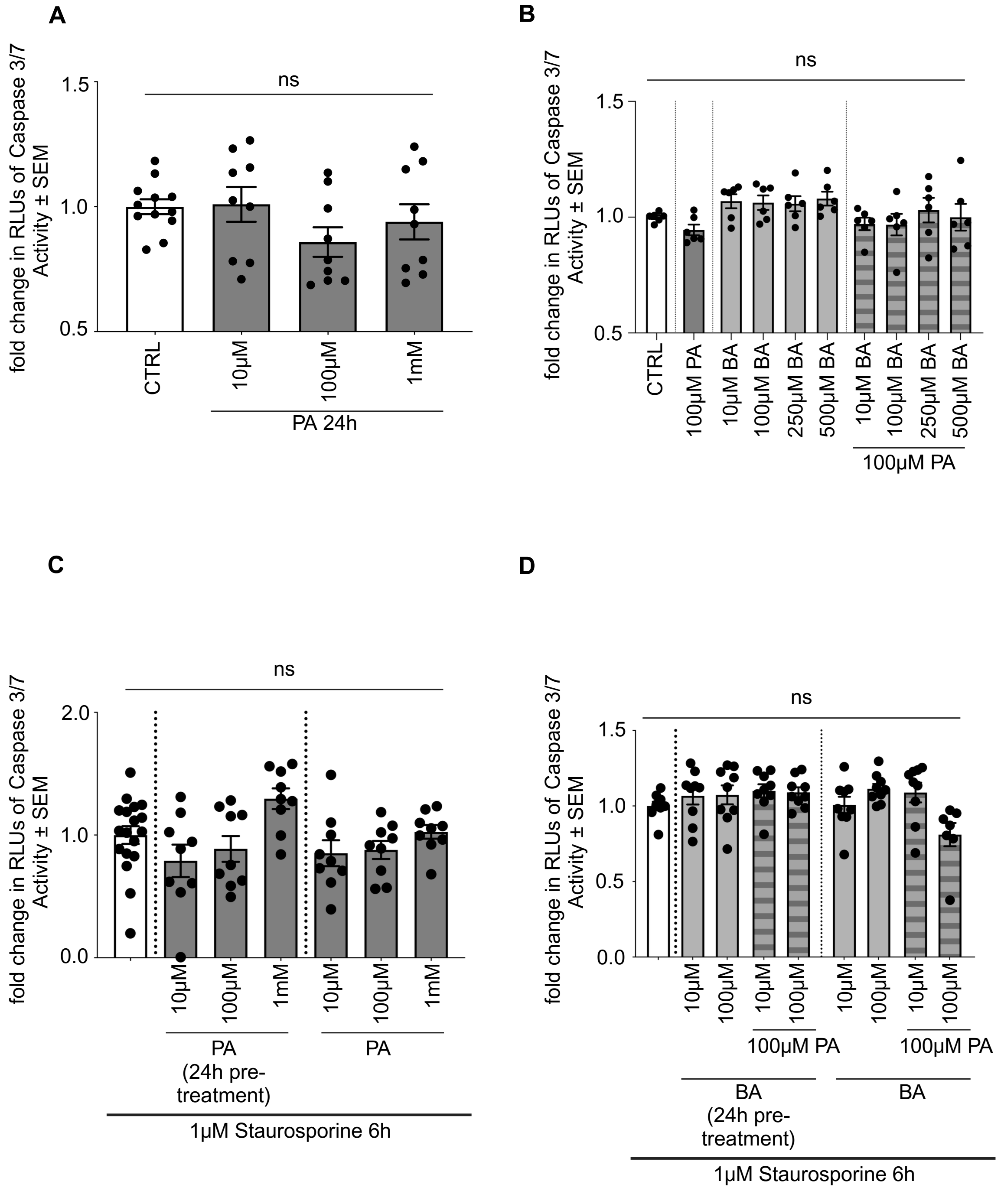

Supplementary Figure 4. iPN apoptosis in the presence of SCFAs. (**A**) Luminescent activity of caspase 3 and 7 activation following cultivation of iPNs in the presence of different PA concentrations for 24 h (CTRL *n* = 12; 10 µM PA *n* = 9, 100 µM PA *n* = 9, 1000 µM PA *n* = 9). (**B**) Luminescent activity assay of caspase 3 and 7 activation following cultivation of iPNs in

the presence of different BA concentrations and in the combined treatment with 100 µM PA (*n* = 6) (**C**) Luminescent activity assay of caspase 3 and 7 activation of iPNs induced by staurosporine treatment for 6 h following 24 h PA pretreatment or in the direct treatment for 6 h (Stauro *n* = 18, 24h 10 µM PA *n* = 9; 24h 100 µM PA *n* = 9; 24h 1000 µM PA *n* = 9; 10 µM PA *n* = 9; 100 µM PA *n* = 9; 1000 µM PA *n* = 9). (**D**) Luminescent activity assay of caspase 3 and 7 activation of iPNs induced by staurosporine treatment for 6 h following BA pretreatment for 24 h, or direct treatment for 6 h, as well as for the combined treatment with 100 µM PA. (*n* = 9; 6h 100 µM BA + 100 µM PA *n* = 7), Data are represented as mean ± SEM and analyzed by using Kruskal-Wallis Test with Dunn's multiple comparison, ns = not significant.

Supplementary Figure 5

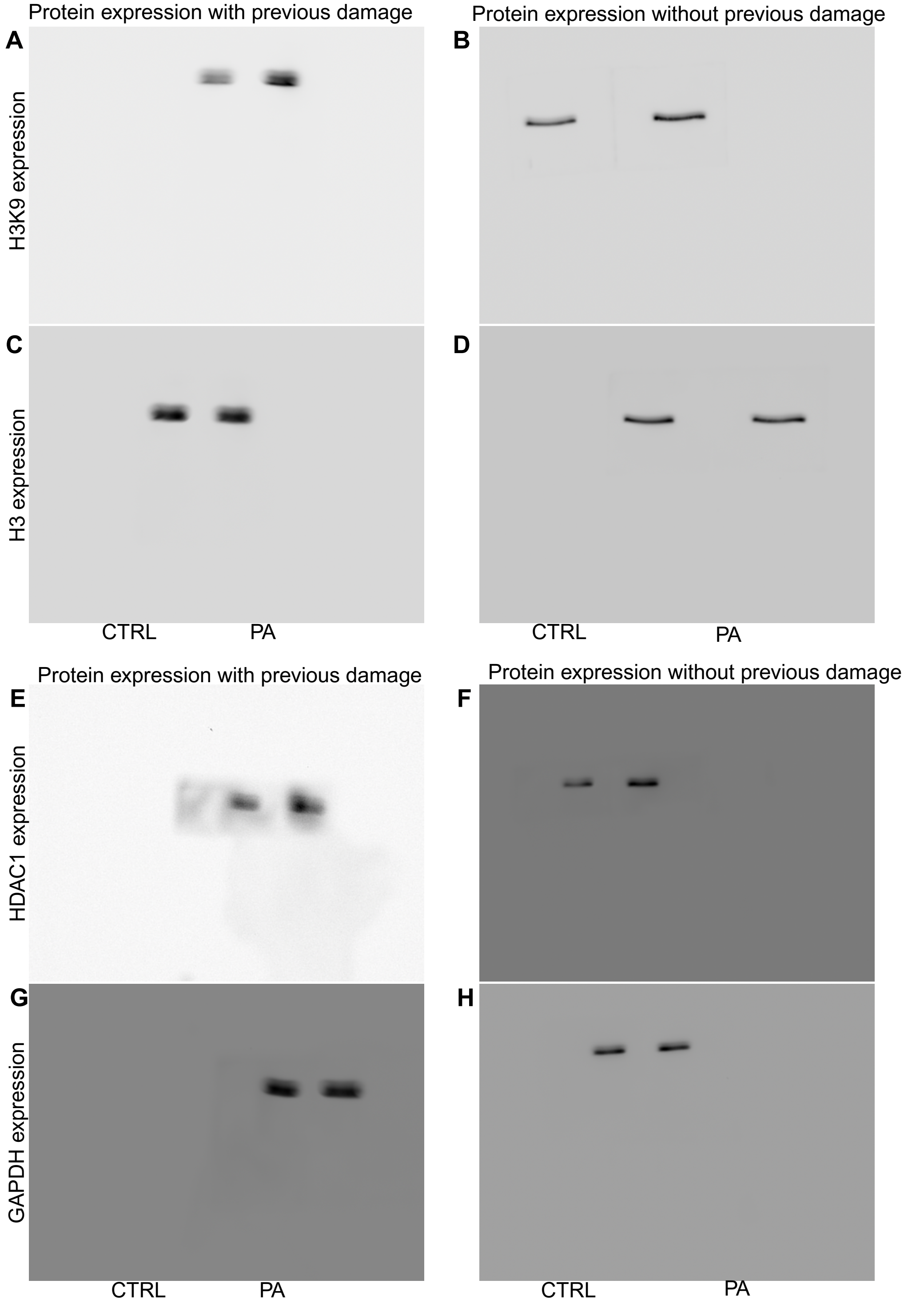

Supplementary Figure 5. Full-size uncropped western blot staining. (A) Full-sized uncropped pictures of membranes stained with anti- H3K9 primary antibody, depicting H3K9

protein expression in iPNs either recovered in neuronal differentiation medium without (left) PA or supplemented with 100μM PA (right), following nocodazole damage. **(B)** Full-sized uncropped pictures of membranes stained with anti- H3K9 primary antibody, depicting H3K9 protein expression in iPNs either cultured in neuronal differentiation medium without PA (left) or supplemented with 100μM PA (right), in the absence of previous damage. **(C)** Full-sized uncropped pictures of membranes stained with anti- H3 primary antibody, depicting H3 protein expression in iPNs either recovered in neuronal differentiation medium without PA (left) or supplemented with 100μM PA (right), following nocodazole damage. **(D)** Full-sized uncropped pictures of membranes stained with anti- H3 primary antibody, depicting H3 protein expression in iPNs either cultured in neuronal differentiation medium without PA (left) or supplemented with 100μM PA (right), in the absence of previous damage. **(E)** Full-sized uncropped pictures of membranes stained with anti- HDAC1 primary antibody, depicting HDAC1 protein expression in iPNs either recovered in neuronal differentiation medium without PA (left) or supplemented with 100μM PA (right), following nocodazole damage. **(F)** Full-sized uncropped pictures of membranes stained with anti- HDAC1 primary antibody, depicting HDAC1 protein expression in iPNs either cultured in neuronal differentiation medium without PA (left) or supplemented with 100μM PA (right), in the absence of previous damage. **(G)** Full-sized uncropped pictures of membranes stained with anti- GAPDH primary antibody, depicting GAPDH protein expression in iPNs either recovered in neuronal differentiation medium without PA (left) or supplemented with 100μM PA (right), following nocodazole damage. **(H)** Full-sized uncropped pictures of membranes stained with anti- GAPDH primary antibody, depicting GAPDH protein expression in iPNs either cultured in neuronal differentiation medium without PA (left) or supplemented with 100μM PA (right), in the absence of previous damage.

Supplementary Figure 6

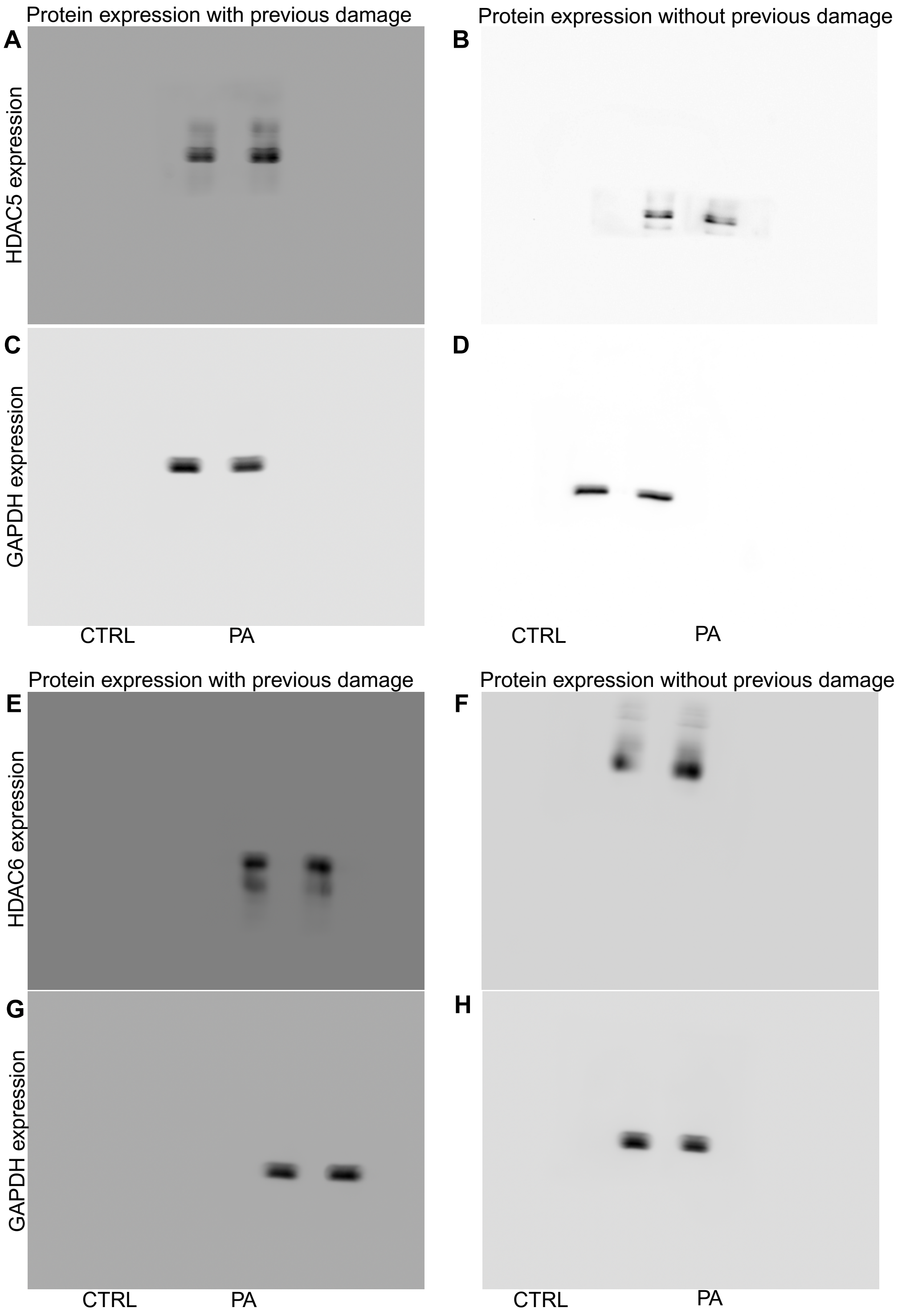

Supplementary Figure 6. Full-size uncropped western blot staining. (A) Full-sized uncropped pictures of membranes stained with anti- HDAC5 primary antibody, depicting HDAC5 protein expression in iPNs either recovered in neuronal differentiation medium without (left) PA or supplemented with 100μM PA (right), following nocodazole damage. **(B)** Full-

sized uncropped pictures of membranes stained with anti- HDAC5 primary antibody, depicting HDAC5 protein expression in iPNs either cultured in neuronal differentiation medium without PA (left) or supplemented with 100μM PA (right), in the absence of previous damage. **(C)** Full-sized uncropped pictures of membranes stained with anti- GAPDH primary antibody, depicting GAPDH protein expression in iPNs either recovered in neuronal differentiation medium without PA (left) or supplemented with 100μM PA (right), following nocodazole damage. **(D)** Full-sized uncropped pictures of membranes stained with anti- GAPDH primary antibody, depicting GAPDH protein expression in iPNs either cultured in neuronal differentiation medium without PA (left) or supplemented with 100μM PA (right), in the absence of previous damage. **(E)** Full-sized uncropped pictures of membranes stained with anti- HDAC6 primary antibody, depicting HDAC6 protein expression in iPNs either recovered in neuronal differentiation medium without PA (left) or supplemented with 100μM PA (right), following nocodazole damage. **(F)** Full-sized uncropped pictures of membranes stained with anti- HDAC 6primary antibody, depicting HDAC6 protein expression in iPNs either cultured in neuronal differentiation medium without PA (left) or supplemented with 100μM PA (right), in the absence of previous damage. **(G)** Full-sized uncropped pictures of membranes stained with anti- GAPDH primary antibody, depicting GAPDH protein expression in iPNs either recovered in neuronal differentiation medium without PA (left) or supplemented with 100μM PA (right), following nocodazole damage. **(H)** Full-sized uncropped pictures of membranes stained with anti- GAPDH primary antibody, depicting GAPDH protein expression in iPNs either cultured in neuronal differentiation medium without PA (left) or supplemented with 100μM PA (right), in the absence of previous damage.

Supplementary Tables

Supplementary table 1 Cluster-robust CR2 SE analyses of neurite regrowth assays.

Figure 1, C

**Characteristic**	**Beta**	**95% CI**[1]	**p-value**
(Intercept)	1.0	1.0, 1.0	<0.001
Condition10µM PA	0.17	0.03, 0.30	0.034
Condition100µM PA	0.26	-0.32, 0.84	0.2
Condition1000µM PA	0.11	-0.18, 0.41	0.2

[1]CI = Confidence Interval; PA = propionic acid

Figure 2, C

**Characteristic**	**Beta**	**95% CI**[1]	**p-value**
(Intercept)	1.0	0.94, 1.1	<0.001
Condition100µM PA	0.27	-0.14, 0.67	0.10
ConditionPTX	0.11	-0.52, 0.75	0.5
ConditionPTX+PA	0.13	-0.24, 0.50	0.2

[1]CI = Confidence Interval; PA = propionic acid; PTX = pertussis toxin

Figure 2, E

**Characteristic**	**Beta**	**95% CI**[1]	**p-value**
(Intercept)	1.0	1.0, 1.0	<0.001
Condition100nm AZD	0.17	-0.58, 0.91	0.2
Condition100nm AZD+PA	0.17	-0.12, 0.46	0.086
Condition50nM AZD	0.00	-0.41, 0.40	>0.9
Condition50nm AZD+PA	0.16	-1.5, 1.8	0.4
ConditionDMSO	0.04	-2.3, 2.4	0.9
ConditionPA	0.19	-1.3, 1.7	0.4

[1]CI = Confidence Interval; DMSO = dimethyl sulfoxide

Figure 6, A

**Characteristic**	**Beta**	**95% CI**[1]	**p-value**
(Intercept)	1.0	1.0, 1.0	<0.001
Condition10µM BA	0.30	0.20, 0.41	0.007
Condition100µM BA	0.14	-0.20, 0.48	0.2
Condition1000µM BA	-0.28	-0.54, -0.01	0.046

Figure 6, A

Characteristic	Beta	95% CI[1]	p-value
Condition250μM BA	-0.04	-0.34, 0.26	0.7
Condition500μM BA	-0.06	-0.50, 0.38	0.6

[1]CI = Confidence Interval; BA = butyric acid

Figure 6, B

Characteristic	Beta	95% CI[1]	p-value
(Intercept)	1.0	1.0, 1.0	<0.001
Condition10μM BA	0.49	-0.28, 1.3	0.11
Condition100μM BA	0.26	-0.10, 0.61	0.088
ConditionPTX	0.05	-0.18, 0.29	0.4
ConditionPTX+10μMBA	-0.07	-0.24, 0.11	0.2
ConditionPTX+100μM BA	0.03	-0.22, 0.27	0.7

[1]CI = Confidence Interval; BA = butyric acid; PTX = pertussis toxin

Figure 7, C

Characteristic	Beta	95% CI[1]	p-value
(Intercept)	1.0	1.0, 1.0	<0.001
Condition10μM BA+PA	0.23	-0.34, 0.80	0.2
Condition100μM BA+ PA	0.30	-0.14, 0.74	0.10
Condition1000μM BA+PA	0.02	-0.63, 0.66	>0.9
Condition250μM BA+PA	0.32	-0.33, 1.0	0.2
Condition500μM BA+PA	0.19	-0.38, 0.77	0.3
ConditionPA	0.33	-0.17, 0.83	0.10

[1]CI = Confidence Interval; BA = butyric acid; PA = propionic acid

Supplementary Figure 1, B

Characteristic	Beta	95% CI[1]	p-value
(Intercept)	1.0	1.0, 1.0	<0.001
ConditionCTRL-recovery	-0.09	-0.54, 0.36	0.5
ConditionNocodazole treatment	-0.42	-0.75, -0.09	0.031

[1]CI = Confidence Interval; CTRL = control condition

Material and Methods

Western Blot

For protein isolation, cells were rinsed once with ice cold DPBS-/-, harvested via a cell scraper after 24 h recovery following nocodazole treatment and collected within a 15 ml falcon tube. Falcons were centrifuged at 450 g for three minutes at 4 °C. For cell lysis, the cell pellet was diluted in 300 µl RIPA buffer (25 mM Tris base, pH 8.0, 150 mM NaCl, 1% (v/v) Triton-X 100, 1% (v/v) DOC, 0.1% (w/v) SDS, 1 mM EDTA, 1x phosphate inhibitor cocktail, 1x protease inhibitor cocktail) and incubated for 30 min on ice. In this time, the pellet was vortexed every 10 min for 30 s. After 30 minutes, the cell pellet was additionally lysed by ultrasound, three times, for 10 seconds. Protein concentration was calculated via the PierceTM BCATM protein Assay (#23225, Thermo Fisher) per manufacture instructions.

For analysis, a total of 10 µg of protein per lane was resolved by SDS-PAGE and transferred onto nitrocellulose membranes. Blocking of unspecific bindings sites was performed in 3% milk powder in TBST for 1 h at RT, followed by primary antibody incubation (rabbit anti histone deacetylase 1, dilution 1:1,000, St. John's Laboratory, #STJ23928; rabbit anti histone deacetylase 5, dilution 1:500, St. John's Laboratory, #STJ29269; rabbit anti histone deacetylase 6, dilution 1:2,000, St. John's Laboratory, #STJ23932; rabbit anti histone H3 (acetyl K9), dilution 1:500, Abcam, #ab10812; mouse anti histone H3, dilution 1:1,00, Abcam, #ab10799; GAPDH, dilution 1:1,000, Santa Cruz, #SC-365062) conducted in 3% milk powder in TBST at 4 °C overnight. The next day, membranes were washed with TBST three times, followed by horseradish peroxidase (HRP)-conjugated secondary antibody (goat anti mouse, IgG, peroxidase conjugated, H+L, dilution 1:10,000, Merck, #AP124P; goat anti rabbit, IgG, peroxidase conjugated, H+L, dilution 1:10,000, Merck, #AP132P) incubation for 2 h at RT. Afterwards, proteins were detected by Immobilon Western HRP Substrate (#WBKLS0500, Merck) detection system per manufactures instructions. Visualization was performed by Bio Rad Chemidoc MP imaging system (Bio Rad) and bands were analyzed by image studio lite (LI-COR Bioscience GmbH).

Caspase 3/7 activity assay

Measurements of iPN caspase 3/7 activation were performed by Caspase Glo® 3/7 Assay (#G8090, Promega). For analysis of anti-apoptotic influence of SCFA treatment, cells were

either preincubated with respective SCFA concentration for 24 h in neuronal medium, or SCFA were supplemented together with 1 μM of the protein kinase A inhibitor staurosporine (#ALX-380-014-C250, Enzo Life Science) for 6 h. Following treatment procedure, medium was removed and replaced by Caspase Glo® 3/7 Assay and treated per manufactures instructions.
